# Engineering IL‐4 resistant proinflammatory human myeloid cells for cancer immunotherapy

**DOI:** 10.1002/1878-0261.70346

**Published:** 2026-10-03

**Authors:** Theresa Barberi, Alan D. Friedman

**Affiliations:** ^1^ Department of Oncology Johns Hopkins University Baltimore MD USA

**Keywords:** human, immunotherapy, macrophages, myeloid cells, NF‐κB p50, STAT6

## Abstract

Adoptive transfer of proinflammatory myeloid cells enhances antitumor T‐cell responses and limits tumor growth in murine models. Previous work identified transcription factors NF‐κB p50 and STAT6 as key regulators whose inactivation enhances the inflammatory potential of murine myeloid cells. Herein, we translate murine findings to human cells and establish methods to generate proinflammatory myeloid cells from human marrow‐derived CD34^+^ hematopoietic stem and progenitor cells (HSPCs). Using CRISPR‐Cas9, we disrupted *NFKB1*, *STAT6*, both *NFKB1* and *STAT6*, or *NFKB1* and *NFKB2* (encoding NF‐κB p52), achieving robust loss of target proteins. We determined conditions for expanding HSPCs and differentiating them into monocytes. To evaluate proinflammatory potential under immune‐suppressive conditions, edited monocytes were differentiated into macrophages, exposed to IL‐4, and analyzed for gene expression. RNA sequencing revealed enhanced proinflammatory pathway activation in STAT6‐ and p50/STAT6‐deficient macrophages, but not in p50‐ or p50/p52‐deficient macrophages. Transcriptomic analysis was validated by measuring protein secretion of a subset of inflammatory mediators. These findings establish a strategy to generate proinflammatory human myeloid cells with potential clinical application in cancer immunotherapy. Future clinical evaluation will be required to determine whether STAT6‐IMC or p50/STAT6‐IMC exhibit superior therapeutic efficacy.

## Graphical abstract

We developed a scalable workflow to generate proinflammatory human myeloid cells. CRISPR/Cas9‐edited CD34^+^ hematopoietic stem and progenitor cells were expanded and differentiated with M‐CSF. Deletion of STAT6 or STAT6/NFKB1 enhanced macrophage proinflammatory gene expression and cytokine secretion in the presence of IL‐4 while maintaining antibody‐mediated tumor‐cell phagocytosis. This platform enables the production of engineered myeloid cells for potential adoptive cancer immunotherapy.

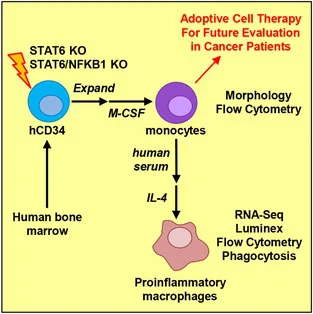

**Abbreviations**
5FU: 5‐fluorouracil
BMDM: bone marrow‐derived macrophages
CFSE: carboxyfluorescein succinimidyl ester
DC: dendritic cell
DEG: differentially expressed genes
FcγR: Fcγ receptor
FL: Flt3 ligand
GM‐CSF: granulocyte‐monocyte colony‐stimulating factor
GMFI: geometric mean fluorescence index
GO: gene ontology
GSEA: gene set enrichment analysis
HSPCs: hematopoietic stem and progenitor cells
ICE: inference of CRISPR editing
IFNγ: interferon γ
IL‐4: interleukin 4
IL‐6: interleukin‐6
IMC: immature myeloid cells
KO: knockout
LLoD: lower limit of detection
LPS: lipopolysaccharide
M‐CSF: monocyte colony‐stimulating factor
MHC: major histocompatibility complex
NES: normalized enrichment score
NLS: nuclear localization signal
NT: non‐targeting
p50: NF‐κB p50
p52: NF‐κB p52
qRT‐PCR: quantitative real‐time PCR
RNA‐Seq: RNA sequencing
RNP: ribonucleoprotein
SCF: stem cell factor
SFEM: serum‐free expansion media
sgRNA: small‐guide RNAs
TAM: tumor‐associated macrophages
TPM: transcripts per million
TPO: thrombopoietin
WT: wild‐type

## 1 Introduction

The tumor microenvironment secretes chemokines that recruit circulating monocytes, along with cytokines such as IL‐4, IL‐10, and TGFβ that polarize them into immunosuppressive tumor‐associated macrophages (TAM). These suppressive TAM impair T‐cell effector function, stimulate angiogenesis, and support metastasis. Proinflammatory TAM, by contrast, enhance antitumor immunity and are associated with favorable clinical outcomes [1, 2]. To date, approaches aimed at shifting macrophages from immunosuppressive to proinflammatory states have largely focused on modulating cytokines and signaling pathways; however, their efficacy is often constrained by tumor‐driven resistance mechanisms, macrophage plasticity, and challenges in achieving durable phenotypic conversion [3]. Targeting key transcription factors that reinforce immunosuppressive macrophage programs offers an alternative strategy to more stably reprogram inflammatory responses at their source, potentially circumventing the resistance mechanisms that limit single‐cytokine approaches. We therefore sought to develop genetically programmed proinflammatory myeloid cells capable of resisting IL‐4‐mediated suppression as a potential platform for adoptive immunotherapy in cancer.

Given the overexpression of transcriptionally repressive p50 homodimers in suppressive TAM [4], we initially focused on NF‐κB p50 (p50) as a key regulator of macrophage polarization. NF‐κB p50 is one of five NF‐κB family members, each containing a Rel‐homology domain that mediates dimerization and DNA binding [5]. RelA (also known as NF‐κB p65 or p65), RelB, and c‐Rel contain C‐terminal transactivation domains, whereas p50 and NF‐κB p52 (p52) lack a transactivation domain but interact with histone deacetylase corepressors [6, 7]. The p50 and p65 subunits are most widely expressed. Under basal conditions, p50 : p65 is sequestered in the cytoplasm by IκB, whereas p50 : p50, which has 60‐fold lower affinity for IκB, can freely enter the nucleus, bind DNA, and repress transcription [7, 8, 9]. Inflammatory stimuli such as TNFα and IL‐1β activate IκB kinase (IKK), triggering IκB phosphorylation, ubiquitination, and degradation, thereby allowing p50 : p65 to enter the nucleus, displace p50 : p50, and activate pro‐inflammatory NF‐κB target genes [5]. In murine myeloid cells lacking p50, a subset of NF‐κB target genes becomes derepressed, producing a macrophage pro‐inflammatory phenotype and dendritic cell (DC) activation [10, 11].

Consistent with this mechanism, we and others have observed that melanoma, fibrosarcoma, glioblastoma, colon cancer, prostate cancer, pancreatic ductal carcinoma, and neuroblastoma tumors grow slower in syngeneic p50^−/−^ mice, or in p50(f/f);Lys‐Cre mice lacking p50 specifically in mature myeloid cells [4, 12, 13, 14, 15]. In these models, TAM lacking p50 manifest increased proinflammatory gene expression, as well as reduced expression of suppressive mRNA and surface markers. These shifts in TAM phenotype are accompanied by increased numbers of total and activated (e.g., IFNγ^+^) CD4^+^ and/or CD8^+^ tumor T cells, and T‐cell depletion in these models accelerates tumor growth and negates the survival benefit conferred by intrinsic myeloid cell activation [4, 12, 13, 14, 15].

To extend these findings toward therapeutic application, we first employed an adoptive cell therapy approach in murine models. Adoptive transfer of immature myeloid cells lacking p50 (p50‐IMC) slows the growth of syngeneic murine prostate cancer, pancreatic ductal carcinoma, and neuroblastoma tumors when administered after a dose of 5‐fluorouracil (5FU) [14, 15]. p50‐IMC are obtained by expansion of hematopoietic progenitors from the bone marrow of p50^−/−^ mice in the presence of stem cell factor (SCF), Flt3 ligand (FL), and thrombopoietin (TPO), cytokines that maintain stem/progenitor cell immaturity, or by similar expansion of *Nfkb1* gene‐edited marrow from wild‐type mice. Cells are then transferred to media with monocyte colony‐stimulating factor (M‐CSF) for one day to generate IMC. We adoptively transfer immature myeloid cells rather than mature macrophages because they retain the ability to develop into both macrophages and DCs within tumors and draining lymph nodes; also, in contrast to macrophages, which largely localize to the liver [16], IMC more effectively reach tumors [14]. 5FU is administered five days prior to p50‐IMC to suppress bone marrow production of competing wild‐type (WT) myeloid cells, to reduce TAM numbers, and to directly kill tumor cells and release tumor neoantigens for presentation to T cells by macrophages and DCs. p50‐IMC increases total and activated tumor T cells, and efficacy is obviated by T‐cell depletion [14, 15].

Despite slower growth, murine prostate cancer, pancreatic ductal carcinoma, and neuroblastoma tumors ultimately progress after 5FU/p50‐IMC therapy [14, 15], prompting us to explore additional transcription factors whose deletion might further enhance IMC efficacy. Given that NF‐κB p52 homodimers also function as transcriptional repressors and are partially homologous to p50, dual deletion of p50 and p52 was a promising strategy. In parallel, we considered STAT6, which activates multiple anti‐inflammatory ‘M2’ genes and represses multiple pro‐inflammatory ‘M1’ genes in murine bone marrow‐derived macrophages [17, 18]. Consistent with this role, breast cancer grows slower in STAT6^−/−^ mice [19, 20], and melanoma and colon cancer grow faster in *Trim24*(f/f);LysM‐Cre mice lacking myeloid cell expression of Trim24, which inhibits STAT6 activity via acetylation [21].

p50‐IMC in immune‐competent murine models provides proof‐of‐concept that targeting a transcription factor in adoptively transferred myeloid cells can limit tumor growth *in vivo*. To advance this strategy toward clinical application, we established a human hematopoietic progenitor platform to evaluate p50 and additional transcription factor targets. Using CRISPR/Cas9‐mediated gene‐editing of human marrow CD34^+^ hematopoietic stem and progenitor cells (HSPCs), we generated myeloid cells lacking p50, STAT6, p50 and p52, or p50 and STAT6. We assessed the expansion capacity of edited CD34^+^ cells and defined the kinetics of CD11b^+^CD14^+^ monocyte accumulation upon transfer to M‐CSF. To examine the phenotype of edited cells under immunosuppressive conditions, monocytes were differentiated into macrophages and polarized with IL‐4. Herein, we demonstrate that loss of STAT6, alone or in combination with p50, promotes proinflammatory gene expression and reduces immunosuppressive gene expression, whereas loss of p50 alone or together with p52 does not. While STAT6‐ and p50/STAT6‐deficient macrophages both adopt a net proinflammatory phenotype in the presence of IL‐4, their transcriptional programs exhibit distinct differences, supporting the need for clinical evaluation to determine which approach performs optimally within the complex tumor microenvironment. Taken together, these findings identify two candidate editing strategies for engineering IL4‐resistant proinflammatory human myeloid cells with therapeutic potential in cancer immunotherapy and provide a framework for their optimal production and clinical application.

## 2 Materials and methods

### 2.1 Cell culture

Human bone marrow was obtained as excess from normal donor harvests for allogeneic marrow transplantation, from March 2019 to July 2026. Written informed consent was obtained from each donor prior to specimen collection, in keeping with the Declaration of Helsinki and under the auspices of a research protocol approved by the Johns Hopkins University Institutional Review Board (protocol IRB00127577). Mononuclear cells were isolated by density gradient centrifugation (Ficoll‐Paque; GE Healthcare Life Sciences, Piscataway, NJ, USA), followed by CD34^+^ cell isolation using a CD34 MicroBead Kit (Miltenyi Biotech, Gaithersburg, MD, USA) in the absence of serum. CD34^+^ cells were cultured in serum‐free Expansion Media, consisting of StemSpan SFEM II (StemCell Technologies, Vancouver, Canada) supplemented with human stem cell factor (SCF, 100 ng·mL^−1^; Peprotech, Cranbury, NJ, USA), FLT3 ligand (FL, 100 ng·mL^−1^; Peprotech), thrombopoietin (TPO, 100 ng·mL^−1^; Peprotech), interleukin‐6 (IL‐6, 100 ng·mL^−1^; Peprotech), SR‐1 (750 nm; Cellagen Technology, San Diego, CA, USA), UM171 (35 nm; ExcellThera, Inc., Montreal, Canada), and antibiotic‐antimycotic (AA). Cells were placed into fresh media twice weekly. To induce myeloid differentiation, expanded cells were cultured in StemSpan SFEM II media supplemented with human monocyte‐colony‐stimulating factor (M‐CSF, 50 ng·mL^−1^; Peprotech) or human granulocyte‐macrophage colony‐stimulating factor (GM‐CSF, 50 ng·mL^−1^; Peprotech). Viable cell numbers were enumerated using a hemocytometer after staining with Trypan Blue dye. To produce macrophages, floating cells expanding in M‐CSF for five days were transferred to Iscove's Modified Dulbecco media (IMDM) with 10% human AB serum (H4522; Sigma, St. Louis, MO, USA), plated at 10^6^ cells per well of a 6‐well dish, and cultured for seven additional days. For M1 or M2 polarization, interferon γ (IFNγ, 50 ng·mL^−1^; Peprotech), or interleukin‐4 (IL‐4, 20 ng·mL^−1^; Peprotech), respectively, were then added. To visualize cell morphology, cells were spun onto glass slides for 5 min at 500× ***g*** and stained with Wright–Giemsa dye. SK‐N‐DZ human neuroblastoma cells (ATCC, Manassas, VA, USA) were maintained in Dulbecco's modified Eagle medium (DMEM) with 10% heat‐inactivated fetal bovine serum (HI‐FBS), nonessential amino acids, and AA.

### 2.2 Gene‐editing

Human CD34^+^ cells were subjected to gene‐editing via nucleofection (Lonza 4D nucleofector), per the Gene Knockout Kit v2 User Manual (Synthego, Redwood City, CA, USA) using SF Nucleofector Kit S (V4XC‐2032, Lonza, Walkersville, MD, USA), *S. pyogenes* Cas9 with two nuclear localization signals (SpCas9 2NLS; Synthego), and the following small‐guide RNAs (sgRNAs) that contain 2′‐O‐methyl groups on the first and last three bases and 3′ phosphorothioate bonds between first four and last three bases (*NFKB1, STAT6, and NFKB2* EZ kits; Synthego):

NFKB1 sg6A: 5′‐AUAUAGAUCUGCAACUAUGU + modified EZ Scaffold (80‐mer), and

NFKB1 sg6C: 5′‐UACCCGACCACCAUGUCCUU + modified EZ Scaffold.

STAT6 sg9A: 5′‐AGAAGAUCAAGCGGUGUGAG + modified EZ Scaffold, and

STAT6 sg9C: 5′‐GCCAGCUUCACACUUGGCCC + modified EZ Scaffold.

NFKB2 sg6A: 5′‐CGAGGGACCAGCCAAGAUCG + modified EZ Scaffold, and

NFKB2 sg6B: 5′‐AGGCGUAGAGGGCACCUACU + modified EZ Scaffold.

The sequence of the nontargeting human control sgRNA (Synthego) is: sgNT: 5′‐GCACUACCAGAGCUAACUCA + modified EZ Scaffold.

The sequence of the modified EZ Scaffold is as follows: 5′‐GUUUUAGAGCUAGAAAUAGCAAGUUAAAAUAAGGCUAGUCCGUUAUCAACUUGAAAAAGUGGCACCGAGUCGGUGCUUUU.

sgRNAs were dissolved in Tris‐EDTA, pH 8.0 to obtain 100 pmol·μL^−1^ stock solutions. Human CD34^+^ cells were nucleofected with a 9 : 1 ratio of sgRNA : Cas9 in a final volume of 30 μL per well of a 16‐well nucleofector strip using Buffer SF (with supplement). For *NFKB1* or *STAT6* gene‐editing, 90 pmol of sgA and 90 pmol of sgC were combined with 20 pmol Cas9. For dual *NFKB1/STAT6* or *NFKB1/NFKB2* gene‐editing, 90 pmol of each of the four required sgRNAs was utilized with 40 pmol Cas9. For the nontargeting control, 360 pmol of sgNT was used with 40 pmol Cas9. After combining sgRNA and Cas9 in Buffer SF as described above, ribonucleoprotein (RNP) complexes were allowed to form for 10 min at room temperature. 7.5 × 10^4^ CD34^+^ cells in 10 μL of Buffer SF were then added to 20 μL RNPs (final volume 30 μL per well), followed by nucleofection using program FS‐100. After allowing 10 min at room temperature for recovery, 70 μL of expansion media was added to each well of the nucleofector strip and the cells were then gently transferred into a tissue culture plate with additional expansion media and cultured at 37 °C in a CO_2_ incubator. Efficiency of gene‐editing was assessed in each experiment by genomic DNA PCR amplification using primers:

NFKB1‐F: 5′‐GGCCTGCTTCTTGGAGTGTA and

NFKB1‐R: 5′‐TCATCCAAATTGGGCCACCT, followed by DNA sequencing using primer:

NFKB1‐Seq: 5′‐CGGGCTGTCTCAAGCAAATC

STAT6‐F: 5′‐CGTTCACACTCTGACCTCCC and

STAT6‐R: 5′‐AGGAAGTGCTTCCGAAGTGT, followed by DNA sequencing using primer:

STAT6‐Seq: 5′‐CTCCATGCTCTTGCTCACC.

NFKB2‐F: 5′‐TGCTACTATGCCCTTGGACCT and

NFKB2‐R: 5′‐TTAGTCACATGCAGGACACCC, followed by DNA sequencing using primer:

NFKB2‐Seq: 5′‐CTTCCTGAGGCACCTTTCCC.

### 2.3 Flow cytometry

Cell analysis was conducted using an LSR II or LSRFortessa flow cytometer (BD Biosciences, Franklin Lakes, NJ, USA). Anti‐CD14‐AF488 was purchased from BD Biosciences. All other antibodies were from BioLegend (San Diego, CA, USA). All antibody staining was preceded by incubation for 30 min at 4 °C with 1 : 1000 Live/Dead Aqua (Invitrogen, Carlsbad, CA, USA) in Hank's Balanced Salt Solution (HBSS), followed by a HBSS wash. Cells were then resuspended in MACS Buffer (PBS, 0.5% BSA, and 2 mm EDTA) and incubated for 15 min at 4 °C with 5 μL FcX block (BioLegend). Cell surface antibodies were then added and incubated for 45 min at 4 °C. Stem/progenitor cells were evaluated using anti‐CD34‐APC and anti‐CD38‐BV421. Monocytes were evaluated using anti‐CD11b‐PE/Cy7, anti‐CD14‐AF488, and anti‐CD11c‐BV650. MCSFR (CSF1R) was analyzed using anti‐MCSFR‐PE‐Cy7. Granulocytes were analyzed using anti‐CD15‐PE and anti‐CD66b‐FITC. B and T lymphoid cells were analyzed using anti‐CD19‐BV421 and anti‐CD3‐AF488. IL4R and IFNγR were analyzed using anti‐IL4Rα‐BV421 and anti‐IFNγR‐PE. FcγR1 and FcγR3 were analyzed using anti‐FcγR1‐APC and anti‐FcγR3‐BUV395. Macrophage MHCI, MHCII, CD80, and CD86 were analyzed using anti‐HLA‐ABC‐AF488, anti‐HLA‐DR‐AF647, anti‐CD80‐PE/Cy7, and anti‐CD86‐BV421. GD2 was detected using anti‐GD2‐APC.

### 2.4 RNA analysis

RNA was prepared using NucleoSpin RNA II, with RNase‐free DNase (Machery‐Nagel, Allentown, PA, USA). First‐strand cDNA was generated from 1 μg total RNA using ImProm‐II reverse transcriptase (Promega, Madison, WI, USA) and an oligo‐dT primer at 42 °C for 1 h. Quantitative real‐time PCR (RT‐PCR) was carried out in triplicate using Radiant LoRox SYBR Green supermix (Alkali Scientific, Fort Lauderdale, FL, USA). Primer pairs, including those for the β‐actin (*ACTB*) internal control, are provided (Table S1). Bulk RNA‐Seq was conducted by the Johns Hopkins Experimental and Computational Genomics Core. In brief, 500 ng of total RNA was used for library construction with the TruSeq Stranded Total RNA Library kit according to the manufacturer's instructions (Illumina, San Diego, CA, USA). Quality and quantity of the resulting cDNA was monitored using the Bioanalyzer High Sensitivity kit (Agilent Technologies, Santa Clara, CA, USA). mRNA libraries were sequenced on an Illumina Novaseq 6000 X instrument using 150 bp paired‐end dual indexed reads and 1% of PhiX control. Depth of coverage was targeted to a total of 50 million reads per library.

### 2.5 Protein analysis

Total cellular proteins were prepared in Laemmli sample buffer and subjected to western blotting using p50 (#3035; Cell Signaling, Danvers, MA, USA), STAT6 (#5397; Cell Signaling), p52 (#4882; Cell Signaling), and β‐actin (A‐15; Millipore‐Sigma, Burlington, MA, USA) antibodies, as described [22]. To analyze secreted proteins, cell supernatants (2 mL) were collected from macrophages obtained in AB serum from gene‐edited human CD34^+^ cells after culture with IL‐4 for 48 or 72 h in 6‐well dishes. Cells cultured with IFNγ (50 ng·mL^−1^) and lipopolysaccharide (LPS, 100 ng·mL^−1^, #L2880; Sigma) served as positive controls. Cell counts (both adherent and floating) from each well were obtained so that protein concentrations could be normalized to the number of underlying cells. Luminex bead‐based immunoassays (Millipore‐Sigma) were performed following validated test methods by the Immune Monitoring Core at Johns Hopkins. Concentrations were determined using five parameter log curve fits (using Bioplex Manager 6.0) with vendor provided standards and quality controls. The HCYTA‐60 K panel (Millipore‐Sigma) was used to detect IFNα2, IFNγ, IL‐1β, IL‐6, IL‐12(p70), CXCL9 (MIG), CXCL10 (IP‐10), TNFα, and CCL5 (RANTES) in the supernatant specimens.

### 2.6 Phagocytosis

For phagocytosis assays, SK‐N‐DZ cells were labeled with carboxyfluorescein succinimidyl ester (CFSE) by incubating cells in PBS, 5% HI‐FBS, 5 μM CFSE (Invitrogen, Carlsbad, CA, USA) at 37 °C for 10 min, followed by washing with DMEM/10% HI‐FBS, and culture at 37 °C with DMEM/10% HI‐FBS for 45 min in an ultra‐low attachment (ULA) plate. CFSE‐labeled SK‐N‐DZ cells were incubated for 45 min at 4 °C in PBS, 0.5% BSA, 2 mm EDTA with 50 μg·mL^−1^ GD2 antibody (Dinutuximab, United Therapeutics Corporation, Silver Spring, MD, USA) or human IgG1 isotype control (Bio‐X‐Cell, Lebanon, NH, USA), followed by washing and resuspension in 4 °C IMDM at 1E6 cells/mL. Macrophages were released from culture dishes using TrypLE, washed, and resuspended in 4 °C IMDM at 1E6 cells/mL. 5E4 macrophages were added to 1E5 CFSE‐labeled SK‐N‐DZ cells in round‐bottomed ULA 96‐well plates and cultured for 3 h at 37 °C, followed by flow cytometry for CFSE and CD11b. Propidium iodide was used to exclude dead cells.

### 2.7 Data and statistical analysis

Gene‐editing efficiency was evaluated using Inference of CRISPR Editing (ICE) analysis (Synthego). ICE analysis provides an Indel Score, quantifying all insertions and deletions.

RNA reads were trimmed with fastp. The data were aligned to the hg38 human reference genome using rsem‐1.3.3, allowing generation of estimated upper quantile normalized posterior means estimate transcripts per million (TPM) relative gene expression values. deseq2 v1.14.1 was used for differential expression analysis, adding the sample pairing information into the analysis; output included the average normalized expression of each gene, the log_2_‐fold change and standard error, the Wald stat value, the Wald test *P*‐value, and Benjamini‐Hochberg adjusted *P*‐value. The R package gplots were used to generate heatmaps of the top 100 most significantly differently expressed genes for each comparison. Gene set enrichment analysis (GSEA) was performed using the R package FGSEA [23] on human Hallmark, KEGG, and Gene Ontology (GO) gene sets from Molecular signatures database (MSigDB) 3.0 [24]. The Wald statistic was used to rank the differentially expressed genes to run the analysis. Enrichment scores, with normalized enrichment scores (NES) and adjusted *P*‐values corrected for multiple hypothesis testing, were obtained for the most significant pathways. The Student's *t*‐test was used to compare flow cytometry and RT‐PCR values.

## 3 Results

### 3.1 Expansion of human bone marrow CD34 ^+^ cells and generation of myeloid cells

Human CD34^+^ HSPCs were isolated from bone marrow obtained as excess material from normal donor harvests for allogeneic transplantation. StemSpan SFEM II media, composed of IMDM supplemented with bovine serum albumin, insulin, and transferrin, combined with FL, SCF, TPO, and IL‐6, supports expansion of human CD34^+^ cells [25]. We assessed CD34^+^ cell expansion in this media alone or following addition of the small molecules SR‐1, UM171, or both (Fig. 1A). SR‐1 promotes CD34^+^ cell expansion via inhibition of the Aryl Hydrocarbon Receptor [26], whereas UM171 enhances expansion by inducing the degradation of the histone Lysine‐specific Demethylase 1 (Lsd1) [27, 28, 29]. Maximal expansion was observed when SR‐1 and UM171 were combined, with approximately threefold more cells evident on Day 30 compared with cultures containing either agent alone, consistent with prior reports [27]. Importantly, the CD34^+^CD38^−^ stem/progenitor cell subset was optimally preserved when both small molecules were present (Fig. 1B). We next examined the contribution of IL‐6 to CD34^+^ cell expansion in cells across four different donors. Removal of IL‐6 resulted in 3.9‐fold and 5.0‐fold reductions in expansion in two donors, while no effect was observed in cells from two additional donors during three weeks in culture (Fig. 1C). Based on these results, SFEM II supplemented with FL, SCF, TPO, IL‐6, SR‐1, and UM171 was used as our Expansion Media for all subsequent experiments.

**Fig. 1:**
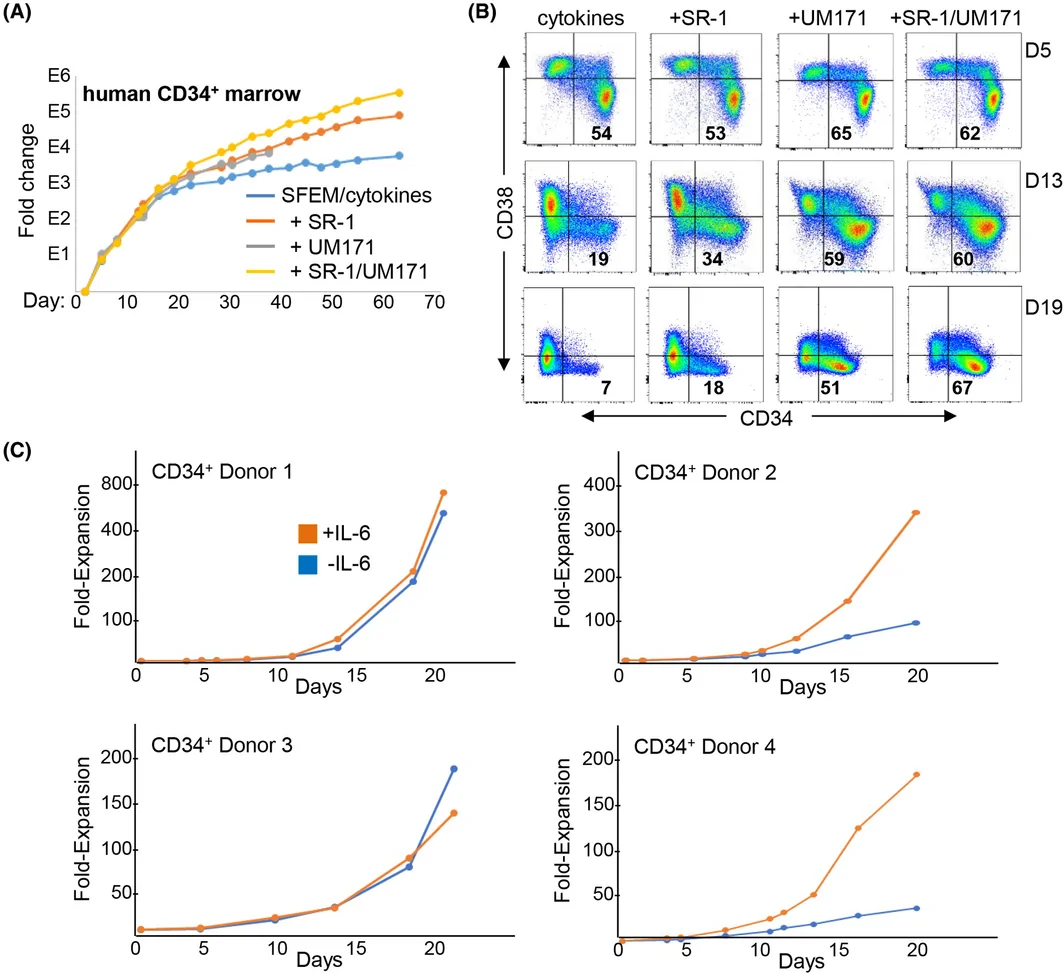
Optimizing human marrow CD34^+^ cell expansion. (A) Expansion of CD34^+^ cells in StemSpan II serum‐free medium (SFEM) with cytokines (SCF, FL, TPO, and IL‐6) alone or with the addition of SR‐1, UM171, or both. E1 = 10^1^; E2 = 10^2^, etc. (*n* = 1). (B) Flow cytometry analysis for CD34 and CD38 on Day 5, Day 13, and Day 19 (D5, D13, D19); (*n* = 1). The proportion of CD34^+^CD38^−^ stem/progenitor cells is shown for each plot. (C) Expansion of wild‐type human CD34^+^ cells from four bone marrow donors was evaluated in SFEM, SCF, FL, TPO, SR‐1, and UM171, each with or without IL‐6 (*n* = 4).

To establish conditions for myeloid cell differentiation, we transferred unedited CD34^+^ cells from Expansion Media into SFEM II supplemented with either M‐CSF or GM‐CSF and cultured them for 5 days. Cells cultured in M‐CSF expanded robustly, whereas those cultured in GM‐CSF exhibited threefold reduced proliferative capacity (Fig. S1A). After 5 days, cells were evaluated for expression of CD11b and the canonical monocyte marker CD14. M‐CSF induced substantial expression of both CD11b and CD14, whereas GM‐CSF resulted in minimal induction of either marker (Fig. S1B). Based on these observations, we selected M‐CSF for differentiation of CD34^+^ progenitors into monocytic cells in all subsequent experiments.

### 3.2 CRISPR/Cas9 sgRNA deletion of *NFKB1* , *NFKB2* , and *STAT6* in human CD34 ^+^ cells

Given the therapeutic efficacy of murine p50‐IMC, we sought to optimize *NFKB1* gene‐editing in human CD34^+^ HSPCs as an initial step prior to targeting additional genes. *NFKB1* was edited using a set of three sgRNAs (sg6A, sg6B, sg6C) designed by Synthego to target exon 6 (of 24 exons total). Upon determining the optimal buffer, sgRNA : Cas9 ratio, and nucleofection program, we achieved 100% editing efficiency (not shown). We subsequently evaluated pairs of these sgRNAs and found that the combination of sg6A and sg6C (sg6AC) consistently yielded 100% indel efficiency across three human donors (Fig. 2A). DNA sequencing confirmed the expected 124‐bp deletion that generates a premature stop codon after Asn97, followed by a frameshift (Figs 2B, S2A). Specifically, fusion of the 5′‐UA from the Tyr98 codon with the 5′‐G from the Asp140 codon creates a UAG stop codon after Asn97. Deconvolution of the primary DNA sequence data indicates the presence of the 124 bp exon 6 deletion in 100% of the edited DNA strands, with 28 ± 2% having additional inserted or deleted base pairs (not shown). Consistent with these genomic alterations, western blot analysis demonstrated a marked reduction in both the p50 protein and its p105 precursor compared with either unedited wild‐type cells or cells nucleofected with a nontargeting (NT) sgRNA (Fig. 2C). Genomic PCR analysis confirmed 99–100% *NFKB1* editing efficiency across all subsequent experiments, both when *NFKB1* was targeted alone and when combined with sgRNAs targeting additional genes.

**Fig. 2:**
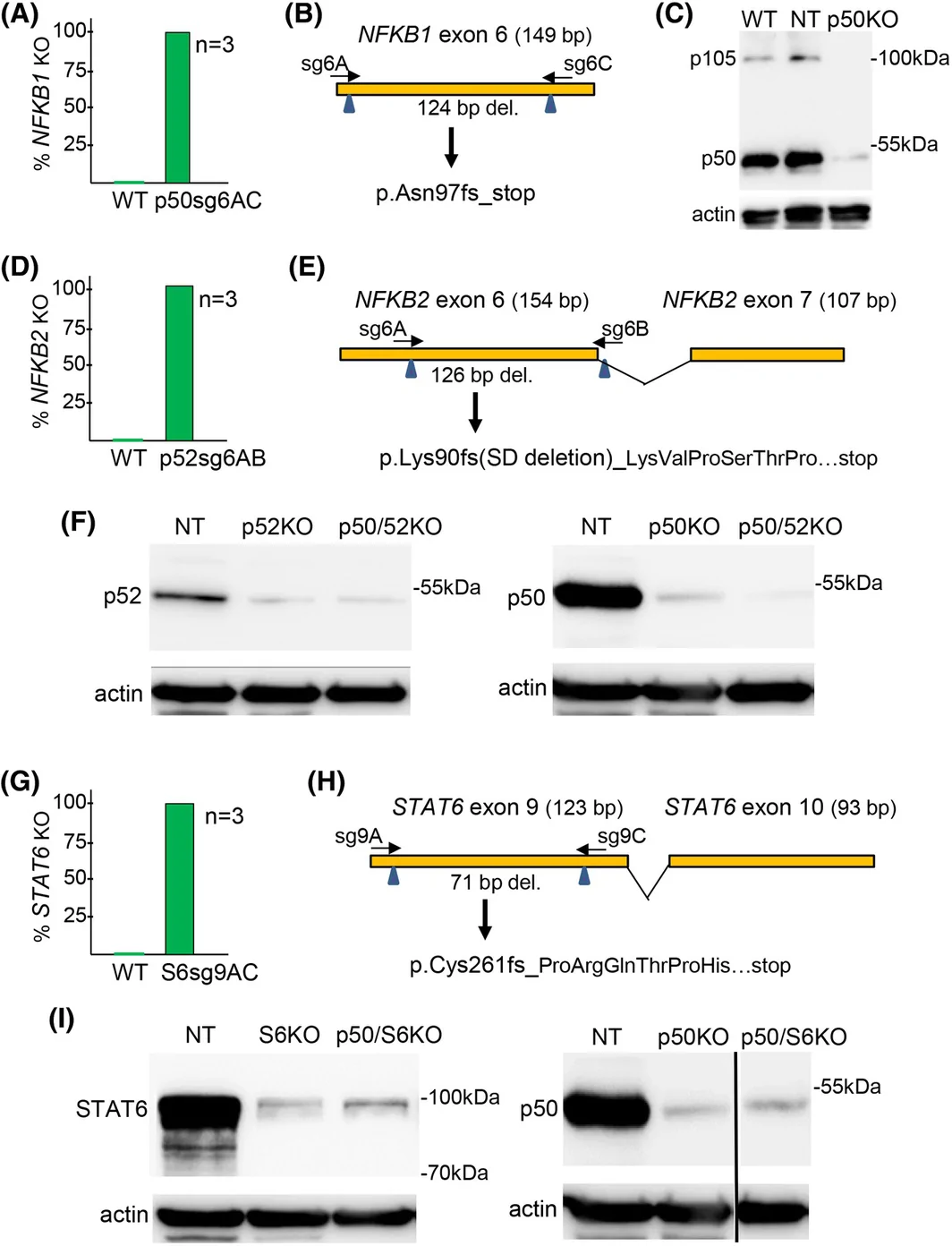
*NFKB1, NFKB2*, *NFKB1 + NFKB2, STAT6*, and *STAT6 + NFKB1* gene‐editing in human marrow CD34^+^ cells. (A) Analysis of *NFKB1* gene‐editing efficiency based on DNA sequencing of PCR products encompassing the deleted segment in human CD34^+^ cells nucleofected with Cas9:sgRNA complexes containing *NFKB1* sg6A + sg6C (p50sg6AC) compared to WT DNA followed by ICE analysis (mean, *n* = 3; SD = 0). (B) Diagram of *NFKB1* gene‐editing. p50sg6AC induces a 124‐bp deletion in exon 6, leading to generation of a stop codon immediately after Asn97, followed by a frameshift. (C) Western blot analysis of p50 and β‐Actin in nonedited WT CD34^+^ cells and cells edited with a nontargeting (NT) sgRNA or with p50sg6AC (p50KO). The location of p105, the precursor of p50, is also indicated (*n* = 1). (D) Analysis of *NFKB2* gene‐editing efficiency based on DNA sequencing of PCR products encompassing the deleted segment in human CD34^+^ cells nucleofected with Cas9:sgRNA complexes containing *NFKB2* sg6A + sg6B (p52sg6AB) compared to WT DNA followed by ICE analysis (mean, *n* = 3; SD = 0). (E) Diagram of *NFKB2* gene‐editing. p52sg6AB induces a 126‐bp deletion encompassing the 3′ end of exon 6 and the first two bps of intron 6, removing the splice donor (SD) site and generating a frameshift expected to produce a STOP codon after 23 aberrant residues. (F) Western blot analysis for p52 (left) and p50 (right) in human CD34^+^ cells after *NFKB1, NFKB2*, or *NFKB1 + NFKB2* gene‐editing, with western blot analysis of β‐Actin as a loading control (*n* = 1). (G) Analysis of *STAT6* gene‐editing efficiency based on DNA sequencing of PCR products encompassing the deleted segment in human CD34^+^ cells nucleofected with Cas9:sgRNA complexes containing *STAT6* sg9A + sg9C (S6sg9AC) compared to WT DNA followed by ICE analysis (mean, *n* = 3; SD = 0). (H) Diagram of *STAT6* gene‐editing. S6sg9AC induces a 71‐bp deletion in exon 9, leading to a frameshift after Cys261, and ultimately a STOP codon after 38 aberrant amino acids. (I) Western blot analysis for STAT6 (left) and p50 (right) in human CD34^+^ cells after *NFKB1, STAT6*, or *NFKB1 + STAT6* gene‐editing, with western blot analysis of β‐Actin as a loading control (*n* = 1). The vertical black line in the p50 blot indicates that the first two and third lanes are non‐adjacent and are from the same image exposure.

To obtain sgRNAs that would efficiently target *NFKB2* and *STAT6*, we utilized the CRISPick [30], CHOPCHOP [31], and InDelphi [32] tools to select and screen several individual sgRNAs and pairs of sgRNAs. We found that paired sgRNAs produced the most efficient and consistent deletions, with *NFKB2* sg6A + sg6B (sg6AB) and *STAT6* sg9A + sg9C (sg9AC) being the most effective.

Editing with *NFKB2* sg6AB resulted in 100% indel efficiency across CD34^+^ cells from three human donors (Fig. 2D). DNA sequencing confirmed the expected 126‐bp deletion that encompasses 124 bp at the 3′ end of exon 6 and the 2 bp GT splice donor site at the 5′ end of intron 6, which creates a frameshift (Figs 2E, S2B). Consequently, Lys90 is predicted to be followed by 23 aberrant amino acids encoded by the retained portion of intron 6, followed by a premature stop codon. Deconvolution of the primary DNA sequence data indicates the presence of the 126 bp exon 6 deletion in 100% of the edited DNA strands, with 35 ± 2% harboring additional inserted or deleted base pairs (not shown). Genomic PCR analysis confirmed 96–100% *NFKB2* gene editing, either alone or in combination with *NFKB1* editing, in each subsequent experiment. Western blot analysis demonstrated efficient loss of p52 and p50 proteins in human marrow CD34^+^ cells after *NFKB2* editing alone or when combined with *NFKB1* editing (Fig. 2F).

*STAT6* sg9AC produced 100% editing efficiency in CD34^+^ cells from three human donors (Fig. 2G), and DNA sequencing confirmed the expected 71‐bp deletion within exon 9 that generates a frameshift, with 38 aberrant C‐terminal amino acids followed by a stop codon in exon 10 (Figs 2H, S2C). Specifically, the in‐frame Cys261 becomes linked to a proline residue rather than glutamate. Deconvolution of the primary DNA sequence data indicates that the 71‐bp deletion is present in 100% of the edited DNA strands, with 7 ± 4% having additional inserted or deleted base pairs (not shown). Genomic PCR analysis confirmed 97–100% *STAT6* gene editing, either alone or in combination with *NFKB1* editing, in each subsequent experiment. Western blot analysis demonstrated highly efficient loss of STAT6 and p50 proteins in human marrow CD34^+^ cells after gene editing of *STAT6* alone or both *NFKB1* and *STAT6* (Fig. 2I).

### 3.3 Expansion and myeloid differentiation of gene‐edited human marrow CD34 ^+^ cells

To determine whether loss of p50, p52, or STAT6 impacts HSPC expansion, we compared proliferation of CD34^+^ cells from three human donors following sgRNA‐mediated deletion of *NFKB1*, *STAT6, NFKB1 + NFKB2, or NFKB1 + STAT6*, with a nontargeting (NT) sgRNA serving as a negative control. Across all three donors, NT‐edited cells proliferated at the highest rate, with p50 or STAT6 single gene deletions expanding ~ twofold less. Cells with dual deletion (p50 + p52 or p50 + STAT6) expanded ~ 1.5‐ to twofold less than single gene‐edited cells (Fig. 3, left). We also observed that delivery of Cas9:sgRNA ribonucleoprotein complexes impaired CD34^+^ proliferation independently of DNA editing, as cells that received the NT sgRNA expanded 3‐ to 5‐fold less than non‐nucleofected, wild‐type (WT) cells (Fig. S3).

**Fig. 3:**
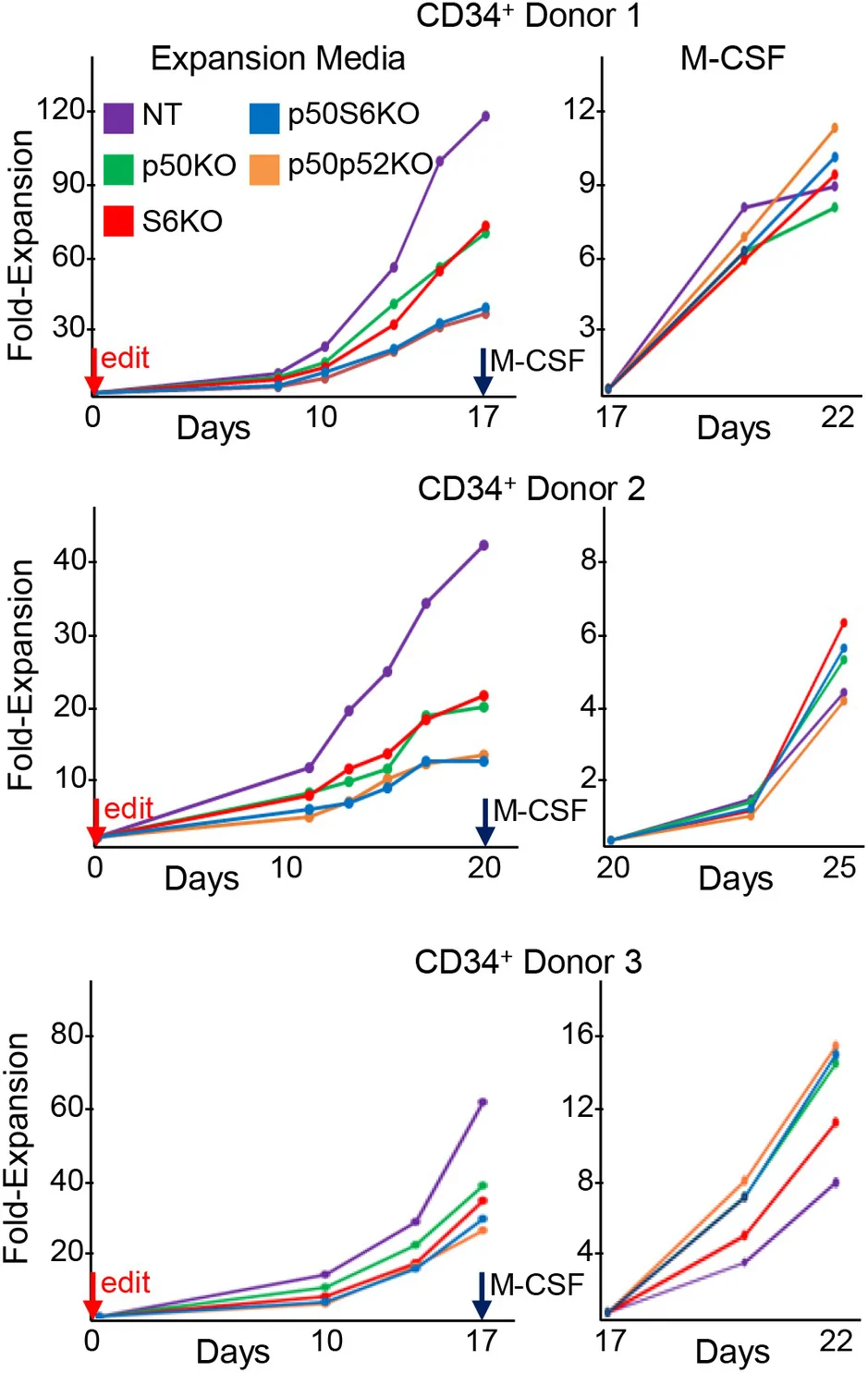
Expansion of human marrow CD34^+^ marrow cells after *NFKB1*, *STAT6*, *NFKB1 + STAT6*, or *NFKB1 + NFKB2* gene‐editing. CD34^+^ cells from Donors 1–3 growing in Expansion Media were subjected to CRISPR/Cas9 gene‐editing with NT, *NFKB1* (p50KO), *STAT6* (S6KO), *NFKB1* + *STAT6* (p50S6KO), or *NFKB1* + *NFKB2* (p50p52KO) sgRNAs (red arrows) and transferred to SFEM II with M‐CSF 17 or 20 days later (blue arrows). Fold‐expansion relative to the cell numbers present on Day 1 in Expansion Media (left) or after transfer to M‐CSF (right) is shown for each marrow donor (*n* = 3).

Having established M‐CSF as the optimal cytokine for monocyte differentiation, we next evaluated M‐CSF receptor expression in our gene‐edited CD34^+^ cells. Receptor expression was comparable across *NFKB1*, *STAT6*, *NFKB1* + *NFKB2*, and *NFKB1* + *STAT6* gene‐edited cells (Fig. S4). We then transferred the edited CD34^+^ cells from Expansion Media into SFEM II supplemented with M‐CSF for 5 days to assess differentiation and expansion. Notably, proliferation differences observed in Expansion Media were no longer evident once transferred to M‐CSF. Instead, *NFKB1, STAT6, NFKB1 + NFKB2*, and *NFKB1 + STAT6* gene‐edited cells expanded at similar rates, indicating that signaling downstream of the M‐CSF receptor remained intact (Fig. 3, right).

We utilized flow cytometry to evaluate the kinetics of CD34^+^ cell differentiation into monocytes during their 5 days in M‐CSF. Representative flow plots are shown, with the mean proportion of CD11b^+^CD14^+^ monocytes on each day indicated for Donors 1–3 (Fig. 4A). Monocytic maturation proceeded at a similar rate for *NT*, *NFKB1*, *STAT6, NFKB1 + NFKB2*, and *NFKB1 + STAT6* gene‐edited cells. No significant differences were observed, with ~ 30% CD11b^+^CD14^+^ monocytes on Day 3 in M‐CSF, ~ 58% on Day 4, and ~ 76% on Day 5. Coincident with development of monocytic cells, the proportion of CD34^+^CD38^−^ stem/progenitor cells decreased, representing ~ 60% of total cells on Days 0–2, ~38% on Day 3, ~ 16% on Day 4, and ~ 3% on Day 5 (Fig. S5). Accordingly, the geometric mean fluorescence index (GMFI) of CD11b and CD14 increased markedly during differentiation, while the GMFI of CD34 among CD34^+^CD38^−^ cells markedly decreased (Fig. S6A–C).

**Fig. 4:**
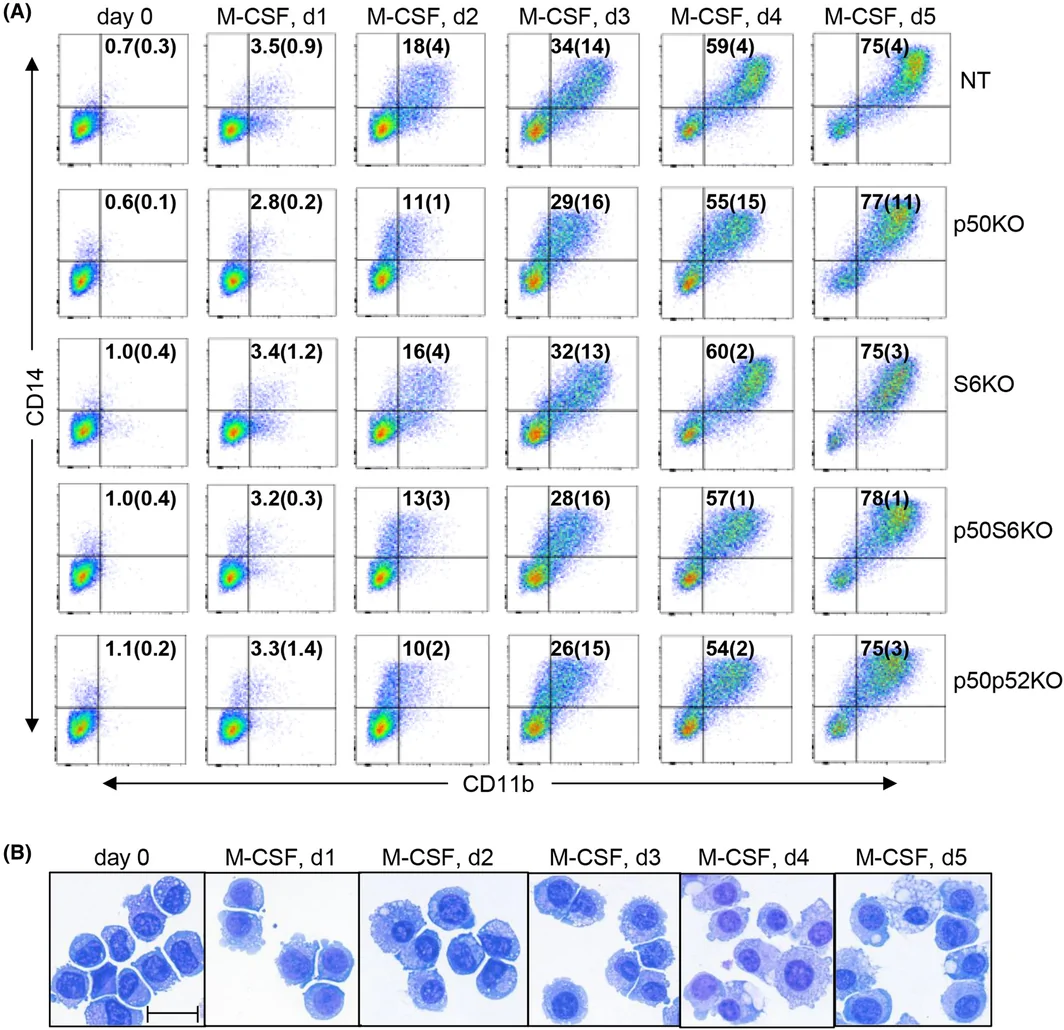
Conversion of expanded and gene‐edited human marrow CD34^+^ cells into immature myeloid cells. (A) CD34^+^ cells from Donors 1–3 growing in Expansion Media were subjected to CRISPR/Cas9 gene‐editing with nontargeting (NT), p50KO, STAT6KO (S6KO), p50 + STAT6 (p50S6KO), and p50 + p52 (p50p52KO) sgRNAs and transferred to SFEM II with M‐CSF. CD11b/CD14 expression was analyzed by flow cytometry beginning at the time of M‐CSF addition (Day 0) and then daily for five days (M‐CSF, d1–d5). Representative flow plots and mean and SD values for the proportion of CD11b^+^CD14^+^ monocytic cells are shown (*n* = 3). (B) Cell morphology was assessed by Wright–Giemsa staining on Day 0 and on Days 1–5 in M‐CSF for each sample analyzed in Part A. Morphologies are shown after NT editing (252X, *n* = 1); these were similar after p50KO, S6KO, or p50S6KO. Scale bar—20 μm.

Although the proportion of CD11b^+^ cells was similar across all edited cells (83–87% on D5), loss of p50 reduced CD11b GMFI ~ twofold on Days 3–5. In contrast, CD14 expression remained consistent between gene‐edited cells, with no significant differences in either the proportion of CD14^+^ cells or CD14 GFMI (Fig. S5). The preservation of CD14 expression suggests that reduced CD11b expression in the absence of p50 does not impair monocytic maturation.

After five days in M‐CSF, most cells remain nonadherent. The nonadherent cells in M‐CSF were cytospun onto glass slides each day and subjected to Wright–Giemsa staining. Representative morphologies for NT cells are shown on each day (Fig. 4B). Cells with increased cytoplasmic volume were evident on Days 4 and 5. Morphologies were similar for NT, *NFKB1*, *STAT6, NFKB1 + STAT6*, or *NFKB1 + NFKB2* edited cells from each donor (not shown).

To further characterize the functional potential of edited human IMC, we evaluated expression of IL‐4 Receptor (IL4R), IFNγ Receptor (IFNγR), Fcγ Receptor 1 (FcγR1), and Fcγ Receptor 3 (FcγR3). The proportions of IL4R^+^ and IFNγR^+^ cells were similar across genotypes (average ~ 77% and ~ 91%, respectively) (Fig. 5A). However, the GMFI of IL4R was approximately two‐fold higher in p50‐, p50/STAT6‐, and p50/p52‐deficient cells compared with NT or STAT6KO cells, potentially influencing downstream IL‐4‐induced gene expression. FcγR1 was present on ~ 96% of NT, *NFKB1, STAT6, NFKB1 + STAT6*, and *NFKB1 + NFKB2* edited cells, whereas FcγR3 was expressed on only ~ 9% of cells from each genotype (Fig. 5B).

**Fig. 5:**
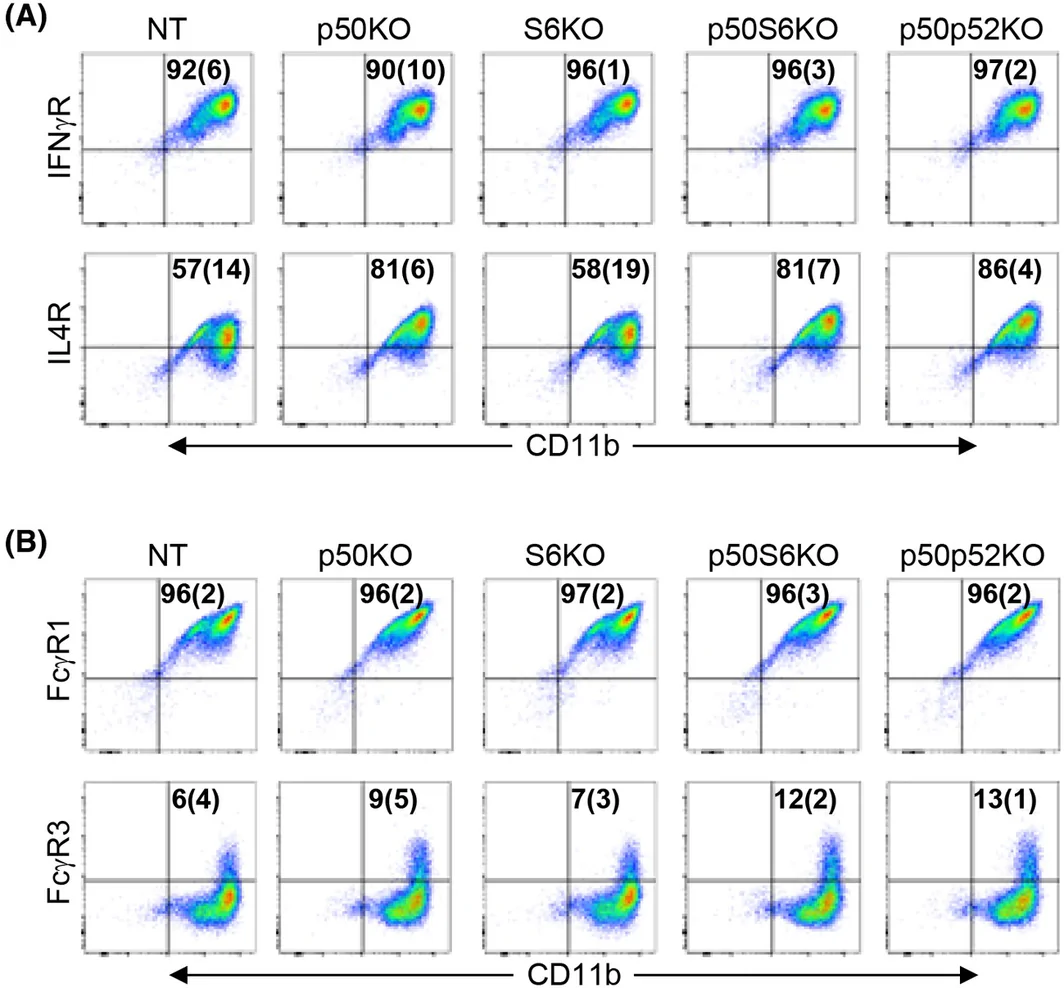
Expression of IL‐4 receptor, IFNγ receptor, and FcγRs in gene‐edited human marrow CD34^+^ cell‐derived immature myeloid cells. (A, B) IL‐4 receptor (IL4R), IFNγ receptor (IFNγR), FcγR1, and FcγR3 were analyzed by flow cytometry after 5 days of M‐CSF, together with CD11b. NT, p50KO, S6KO, p50S6KO, and p50p52KO cells were evaluated. Representative flow plots, mean values, and SD values for the proportion of CD11b^+^IL4R^+^, CD11b^+^IFNγR^+^, CD11b^+^FcγR1^+^, and CD11b^+^FcγR3^+^ cells are shown for the same three donors analyzed in Fig. 4 (*n* = 3).

To assess whether our culture conditions induced differentiation into additional hematopoietic lineages, we evaluated expression of lymphoid, granulocyte, and erythroid markers in gene‐edited HSPCs. On Day 0, prior to transfer to M‐CSF, none of the cells expressed the B‐cell marker CD19 or the T‐cell marker CD3, ~ 4% co‐expressed the granulocyte markers CD15 and CD66b, and ~ 4% expressed the erythroid marker CD235a. CD15, CD66b, and CD235a were expressed at a low GMFI compared to the marrow‐derived mononuclear cell control, suggesting that the few positive events may not represent true granulocyte or erythroid cells. In addition, the signal observed in the CD15^+^CD66b^+^ quadrants likely represents a tail of autofluorescence. After five days in M‐CSF, the proportion of cells expressing these low‐level markers decreased to 0–2% (Fig. S7A–C), except for CD15, which was present on 8–20% of total cells. These CD15^+^ cells also exhibited low CD11b expression, consistent with a minor population of immature granulocytes.

Taken together, these data demonstrate that culture of *NFKB1‐*, *STAT6‐, NFKB1 + NFKB2‐*, or *NFKB1 + STAT6*‐edited human CD34^+^ cells in M‐CSF for 4 or 5 days yields IMC with a similar phenotype to murine p50‐IMC obtained after one day in M‐CSF, which provides therapeutic efficacy in tumor‐bearing mice [14]. Additionally, edited human IMC retain expression of several key receptors, with IL4R and IFNγR enabling responses to cues in the tumor microenvironment, and FcγR1 supporting future efforts to combine IMC with antibodies to help direct them to tumors.

### 3.4 Absence of STAT6 favors proinflammatory gene expression by macrophages in IL‐4

Adoptively transferred murine p50‐IMC develop predominantly into macrophages within tumors [14]. To evaluate the potential impact of transcription factor gene‐editing on human macrophage gene expression, we first established an *in vitro* protocol to successfully generate mature macrophages. Human marrow CD34^+^ cells were initially cultured in SFEM II media with M‐CSF for seven days, mimicking an approach effective for generating murine bone marrow‐derived macrophages (BMDM). Unlike murine cells, a high proportion of human cells remained nonadherent, perhaps reflective of a lack of complete maturation. We therefore adjusted our cell culture protocol based on an approach that allows generation of adherent macrophages from human blood monocytes [33]. After human marrow CD34^+^ cells were expanded in Expansion Media, they were placed in SFEM II media with M‐CSF for five days to generate monocytes, followed by the transfer of floating cells into IMDM with 10% human AB serum for seven days. This strategy produced uniformly adherent macrophages.

After establishing a method to generate adherent human macrophages, we next evaluated changes in gene expression resulting from loss of p50, STAT6, p50/p52, and p50/STAT6. CD34^+^ bone marrow cells were obtained from three human donors (D1–D3) and subjected to NT, *NFKB1, STAT6, NFKB1 + NFKB2*, or *NFKB1 + STAT6* gene‐editing. Edited cells were expanded, cultured in M‐CSF for five days, and then differentiated into macrophages in AB serum. To model conditions characteristic of the immunosuppressive tumor microenvironment, macrophages were exposed to IL‐4 for an additional 24 h, followed by RNA isolation and bulk RNA sequencing analysis.

Normalized gene expression data for all genes analyzed are provided (Table S2). To assess the global transcriptional impact of transcription factor loss, gene expression profiles of NT and gene‐edited macrophages were compared. Heatmaps for the top 100 differentially expressed genes (DEGs) revealed distinct transcriptional programs in macrophages lacking STAT6 or p50/STAT6 compared with NT controls, whereas macrophages lacking p50 or p50/p52 exhibited more modest alterations under IL‐4‐polarizing conditions (Fig. 6A–D, left).

**Fig. 6:**
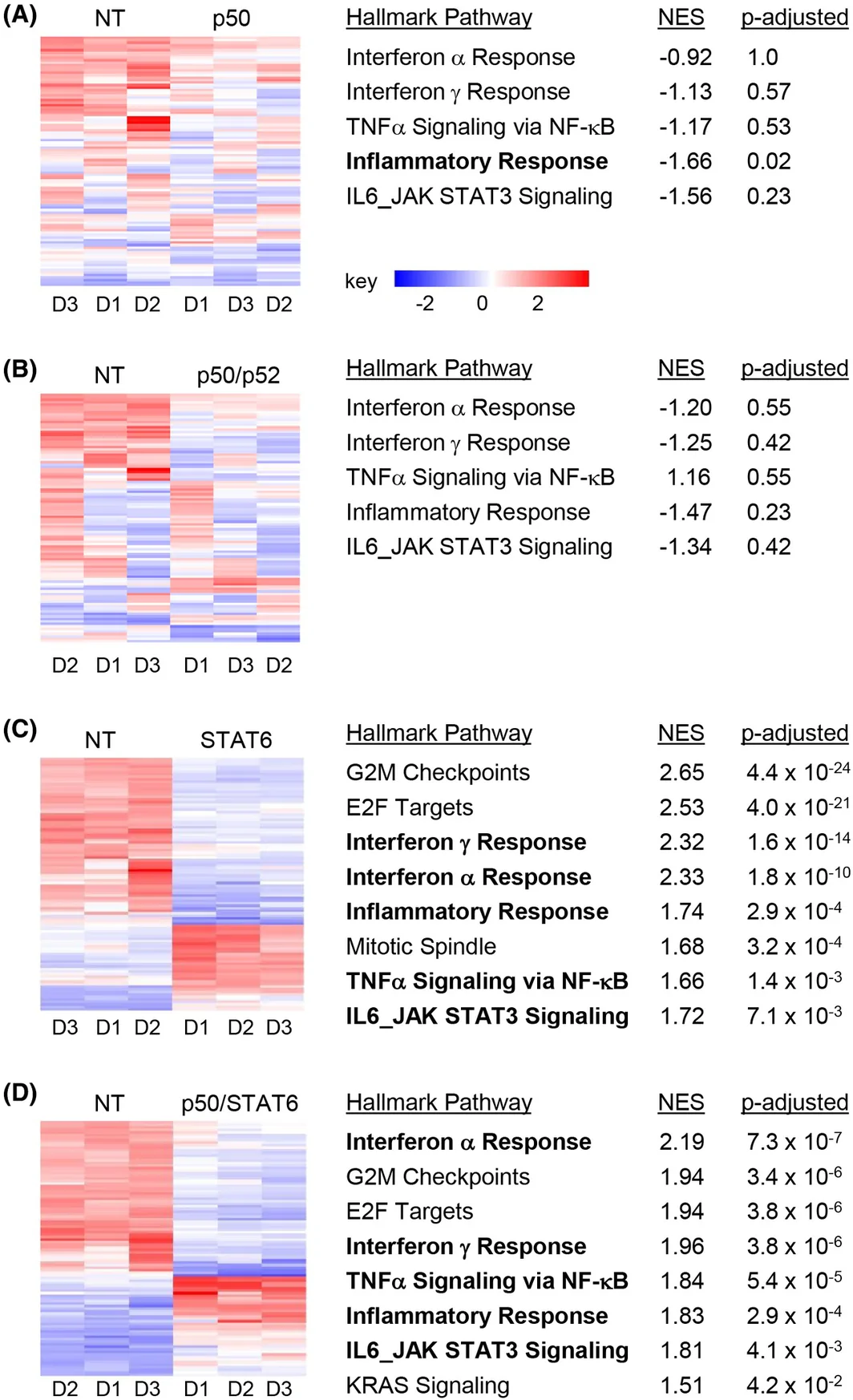
Gene expression analysis of human CD34^+^ cell‐derived macrophages in IL‐4, obtained after NT, *NFKB1, NFKB1 + NFKB2*, *STAT6*, or *NFKB1 + STAT6* gene‐editing. (A–D) Heatmaps of the top 100 differentially expressed genes between NT and transcription factor‐edited macrophages from human donors D1–D3 are shown, along with the top Hallmark pathways activated or repressed in the absence of p50, p50 + p52, STAT6, or p50 + STAT6. NES, normalized enrichment score. Significantly altered pro‐inflammatory pathways appear in bold text.

Given the pronounced transcriptional changes observed in STAT6‐ and p50/STAT6‐deficient macrophages, we next performed gene set enrichment analysis (GSEA). Consistent with the heatmap analysis of the top 100 DEGs, pathway‐level enrichment revealed minimal transcriptional reprogramming in p50‐ or p50/p52‐deficient macrophages. In p50‐deficient cells, the only significantly altered Hallmark pathway was the Inflammatory Response, which was decreased rather than increased (Fig. 6A,B, right). In contrast, loss of STAT6 or combined p50/STAT6 deficiency resulted in broad and statistically significant activation of Hallmark inflammatory pathways, including the Interferon α Response, Interferon γ Response, TNFα Signaling via NF‐κB, Inflammatory Response, and IL6_JAK_STAT3 Signaling (Fig. 6C,D, right).

KEGG pathway analysis yielded results consistent with the Hallmark pathway analysis. No significant changes were observed in p50‐deficient macrophages, and p50/p52‐deficient macrophages showed decreased expression of inflammation‐related pathways. STAT6‐deficient macrophages exhibited increased expression of KEGG inflammatory pathways, including Toll‐Like Receptor Signaling, Cytosolic DNA Sensing, NOD‐Like Receptor Signaling, and RIG1‐Like Receptor Signaling, whereas dual p50/STAT6 deficiency showed enrichment only for Toll‐Like Receptor Signaling (Table S3, left). Consistent with KEGG analysis, Gene Ontology (GO) pathway enrichment showed no significant changes in p50‐deficient cells. In p50/p52‐deficient macrophages, GO pathways related to Antigen Presentation MHCII, Inflammatory Response, and Immune Response were significantly reduced. p50/STAT6‐deficient macrophages also exhibited decreased Antigen Presentation by MHCII, as well as decreased Antigen Presentation by MHCI, whereas single‐deficient cells did not, indicating that the combined loss of p50 and STAT6 may impair antigen presentation and the capacity to activate T cells (Table S3, right).

Alongside inflammatory reprogramming, STAT6‐ and p50/STAT6‐deficient macrophages exhibited enrichment of cell cycle–associated transcriptional programs. Hallmark pathways related to cell cycle progression, including G2M Checkpoint, E2F Targets, and Mitotic Spindle, were significantly enriched in STAT6‐deficient cells, with G2M Checkpoint and E2F Targets also enriched following combined loss of p50 and STAT6. Consistent with these findings, KEGG analysis indicated upregulation of Cell Cycle and DNA Replication pathways in STAT6‐deficient macrophages, while GO pathway analysis demonstrated increased enrichment of the Mitotic Cell Cycle pathway in both STAT6‐ and p50/STAT6‐deficient cells (Table S3). STAT6 has been shown in some contexts to promote cell cycle progression through downmodulation of the cyclin‐dependent kinase inhibitors p21 and p27 [34, 35], providing a potential mechanistic basis for the cell cycle–associated pathway enrichment observed in STAT6‐deficient macrophages.

Because canonical inflammatory pathways identified by GSEA include both pro‐ and anti‐inflammatory genes, we next assessed whether transcription factor loss specifically promoted a proinflammatory macrophage phenotype in the presence of IL‐4. To this end, we assembled a proinflammatory gene set based on a published analysis by Smyth *et al*., in which 260 human monocyte‐derived macrophage RNA‐seq datasets were used to characterize gene expression patterns under commonly used *in vitro* polarization conditions [36]. We focused on 155 genes (Fig. S8A), designated as the Proinflammatory M1 Gene Set, that were increased in IFNγ‐, LPS‐, or IFNγ/LPS‐stimulated human M1 macrophages compared with unstimulated M0 macrophages, found in proinflammatory pathways upon Ingenuity Pathway Analysis (Fig. S8B), and absent from the IL‐4, IL‐10, or IL‐13 stimulated M2 macrophage gene sets [36]. In agreement with prior data obtained with murine macrophages [18], GSEA using the Proinflammatory M1 Gene Set revealed strong enrichment in STAT6‐deficient human macrophages compared with NT controls (NES = 2.29), confirming induction of a proinflammatory transcriptional program despite IL‐4 exposure. p50/STAT6‐deficient human macrophages were similarly enriched for the M1 Gene Set relative to NT controls (NES = 2.20); however, direct comparison of p50/STAT6‐ and STAT6‐deficient macrophages revealed a reduced enrichment score (NES = −1.42), indicating that additional loss of p50 partially attenuates the M1‐associated transcriptional program induced by STAT6 deletion alone (Fig. 7A).

**Fig. 7:**
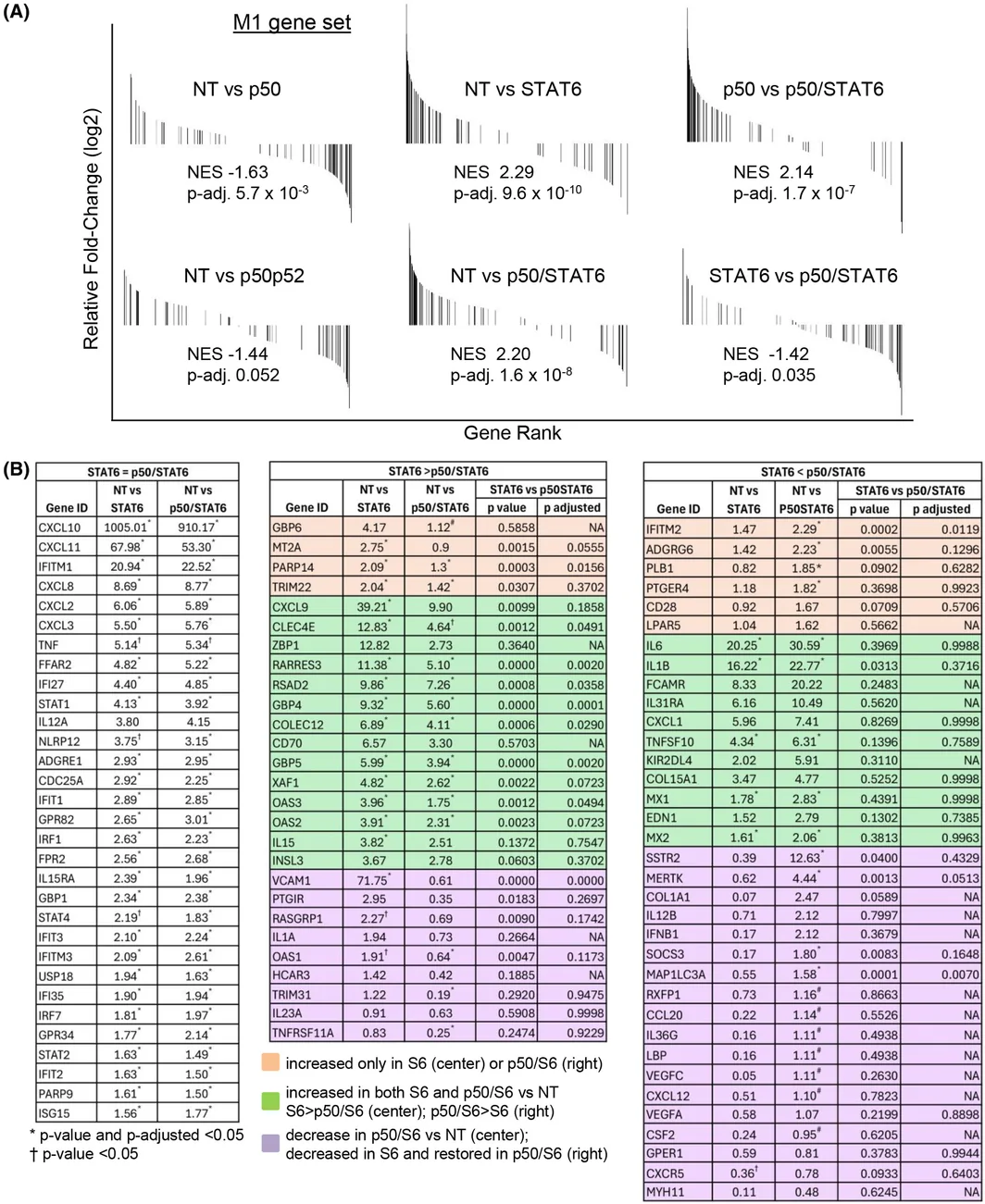
Proinflammatory gene expression in *NFKB1* + *STAT6* versus *STAT6* gene‐editing human CD34^+^ cell‐derived macrophages in IL‐4. (A) The Proinflammatory M1 Gene Set (*n* = 155) was subjected to GSEA using our RNA‐Seq data. Plots of Relative Fold‐Change (log_2_) versus Gene Rank and NES values and their adjusted *P*‐values for six pairwise comparisons are shown, obtained using the R package FGSEA [23]. (B) Members of the M1 Gene Set for which absence of STAT6 or p50 + STAT6 led to a similar increase in expression compared to the NT control in our RNA‐Seq data set (left), those that exhibited greater expression in the absence of STAT6 than in the absence of both p50 and STAT6 (center), and those that exhibited greater expression in the absence of both STAT6 and p50 than in the absence of STAT6 alone (right). Only M1 Gene Set RNAs that were increased at least 1.5‐fold or reduced at least 0.75‐fold in STAT6‐ or p50/STAT6‐deficient macrophages compared to NT controls (*n* = 93) are included in these tables. *P‐value and *p*‐adjusted both < 0.05; ^†^only *P*‐value < 0.05; ^#^ *P*‐value and *p*‐adjusted listed as Not Applicable (NA) vs NT in the RNA‐Seq analysis (due to low expression or large variability) – for these samples *P*‐values comparing STAT6 and p50/STAT6 samples are shown.

To characterize this observed attenuation in M1 gene set enrichment, we compared the fold changes of all 155 members of the Proinflammatory M1 Gene Set between NT and STAT6‐deficient macrophages (NT vs STAT6) and between NT and p50/STAT6‐deficient macrophages (NT vs p50/STAT6) and identified 93 transcripts whose expression was increased by more than 1.5‐fold or was reduced by at least 0.75‐fold due to absence of either STAT6 or both p50 and STAT6. Given the broad transcriptional impact of transcription factor deletion and the small size of our M1 Gene Set, both nominal *P*‐values and p‐adjusted values are reported to highlight potentially meaningful trends that do not meet the false discovery rate threshold applied for adjusted values. Consistent with published murine macrophage data [18], STAT6 deletion significantly increased expression (*P* < 0.05) of 52 of the M1 Gene Set members. Among the 93 M1 Gene Set transcripts with altered expression levels, 31 transcripts were similarly increased in both STAT6‐ and p50/STAT6‐deficient cells relative to NT controls (Fig. 7B, left), 27 reached maximal induction in STAT6‐deficient macrophages (Fig. 7B, center), and 35 were maximally induced in p50/STAT6‐deficient cells (Fig. 7B, right). Within the latter two groups, subsets of transcripts were uniquely upregulated in one knockout, upregulated in both but to different extents, or decreased in one knockout and restored or further increased in the other. Taken together, these data indicate that the reduced M1 gene set enrichment score of p50/STAT6‐deficient macrophages compared to STAT6‐deficient macrophages reflects selective attenuation and restoration of discrete gene subsets rather than broad suppression of proinflammatory transcriptional programs.

To assess the impact of transcription factor deletion on IL‐4‐mediated immunosuppressive programs, we assembled and evaluated an M2 Gene Set (Fig. S8C) comprised of 18 IL‐4‐induced genes, also identified by Smyth et al. [36]. Fifteen of the 18 transcripts were significantly decreased in both STAT6‐ and p50/STAT6‐deficient macrophages, with one transcript being decreased only in STAT6‐deficient cells and two transcripts being decreased only in p50/STAT6‐deficient cells (Fig. S9A). The observed effects of STAT6 KO on M2 gene expression are consistent with prior data obtained with murine macrophages lacking STAT6 [17, 18].

Collectively, RNA sequencing results demonstrate that our gene‐editing strategy produces a transcriptional shift that confers resistance to IL‐4‐induced immunosuppressive programs while promoting proinflammatory gene expression. These data indicate that both STAT6‐ and p50/STAT6‐deficient macrophages adopt a net proinflammatory transcriptional profile; however, the observed differences in proinflammatory gene expression and predicted antigen presentation pathways warranted further evaluation at the protein and functional levels.

### 3.5 Targeted validation and functional assessment of gene‐edited macrophages

We next sought to independently validate selected RNA sequencing findings at the transcript and protein levels and to determine whether gene editing altered key macrophage phenotypic and functional properties. CD34^+^ marrow cells from the same three human donors used for RNA‐Seq analysis were subjected to NT, *NFKB1, STAT6*, or *NFKB1 + STAT6* gene‐editing, followed by expansion, culture in M‐CSF for five days, and conversion to macrophages in serum AB for 7 days. IL‐4 was then added to macrophages for an additional 24 h before RNA lysates were collected. Five proinflammatory transcripts (IL‐6, CXCL10, TNFα, IL1b, and IL‐12b) were assessed by qRT‐PCR (Fig. 8A). Expression patterns closely recapitulated the RNA‐seq results, with *IL6*, *TNFα*, and *IL1β* showing highest expression in p50/STAT6‐deficient macrophages, *IL12α* peaking in p50‐deficient cells, and *CXCL10* being robustly induced (> 250‐fold) in both STAT6‐ and p50/STAT6‐deficient macrophages. We also evaluated five IL‐4–induced suppressive transcripts (*ALOX15*, *CD206*, *CCL13*, *CCL18*, and *CCL22*), which were markedly downregulated in both STAT6‐ and p50/STAT6‐deficient macrophages, consistent with the RNA‐seq data (Fig. S9B).

**Fig. 8:**
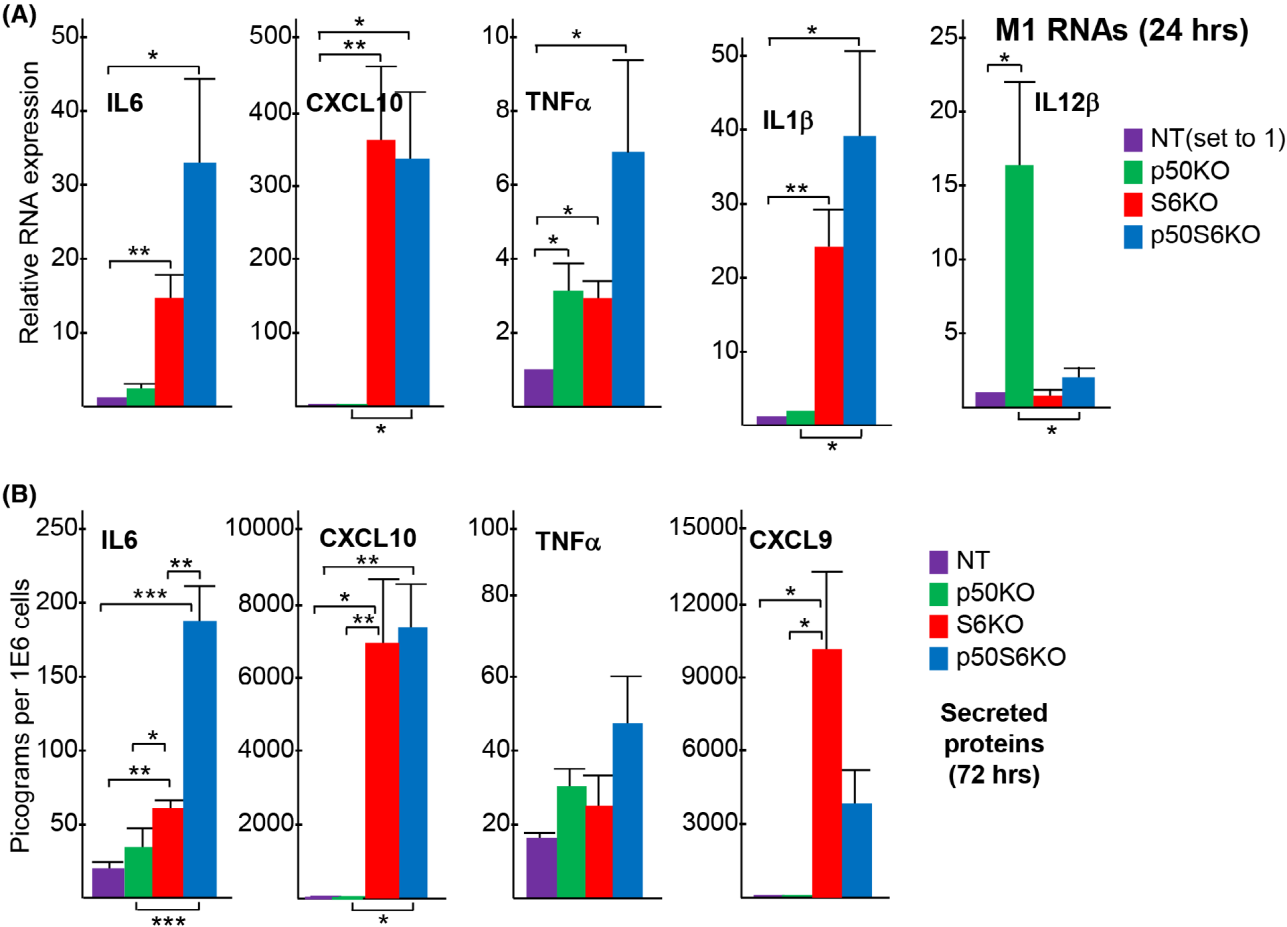
Secreted proinflammatory cytokine/chemokines protein and RNA expression by human CD34^+^ cell‐derived macrophages in IL‐4, obtained after NT, *NFKB1, STAT6*, or *NFKB1 + STAT6* gene‐editing. (A) Quantitative RT‐PCR for indicated RNAs was conducted using the RNAs prepared for RNA‐Seq and an additional sample (*n* = 4), 24 h after IL‐4 addition. Mean values and SDs are shown for each culture and gene‐editing condition. Relative RNA expression levels, normalized to *ACTB*, are shown, with the value in NT conditions set to 1.0. (B) The Luminex assay was used to determine the concentrations of IL‐6, CXCL10, TNFα, and CXCL9 from the three human donors (D1, D2, and D3) used to obtain the RNA‐Seq data described in Fig. 6. Protein concentrations were normalized to total cell numbers enumerated in parallel wells. Data presented were obtained at 72 h after addition of IL‐4. Mean values and SDs are shown for each culture and gene‐editing condition; (*n* = 3). Two of the NT and one of the p50 and STAT6 values were below the TNFα assay lower limit of detection (LLoD), and each of the CXCL9 NT and p50 values were below the CXCL9 assay LLoD; the lower limit values for these samples were used for statistical analysis. *P < 0.05; **P < 0.01; ***P < 0.001, as determined using the Student's *t*‐test.

To determine whether selected transcriptional changes were reflected at the protein level, macrophage supernatants were subjected to Luminex assays to detect proinflammatory mediators IL‐6, CXCL10, TNFα, CXCL9, IL‐1β, IL‐12(p70), CCL5, IFNα2, and IFNγ. After gene‐editing, expansion, culture in M‐CSF, and differentiation to macrophages in serum AB, cells from the same three human donors were polarized with IL‐4 for 72 h. NT macrophages were also cultured in IFNγ and LPS to provide a positive control for protein detection. Values obtained in the Luminex assay were normalized to the number of cells per well (Fig. 8B). IL‐1β, IL‐12(p70), CCL5, and IFNα2 were not detected above the assay's lower limit of detection (LLoD) in any of the IL‐4 samples, although they were detected in the positive control cultures (data not shown). IFNγ expression was not affected by absence of p50, STAT6, or p50 + STAT6 (data not shown). Paralleling RNA‐seq and qRT‐PCR data, the mean IL‐6 secreted per cell among the three donors was increased 2.8‐fold in the absence of STAT6 and 8.3‐fold when both p50 and STAT6 were absent, but was not significantly affected by the absence of p50 alone. CXCL10 secretion was similarly increased (> 130‐fold) in the absence of STAT6 and in the absence of both p50 and STAT6, but was not impacted by deletion of p50 alone.

For both TNFα and CXCL9, some but not all of the samples were below the assay's LLoD. Each analyte evaluated has a unique LLoD, and we assigned that cutoff value to the undetected samples to enable interpretation of the samples that were within the detectable range. Specifically, TNFα was below the LLoD for two NT, one p50, and one STAT6 sample; when those 4 samples were assigned the LLoD cutoff value for the purpose of analysis, we determined a minimum 1.5‐ to twofold increase in TNFα secretion in the absence of p50, STAT6, or p50 + STAT6 compared with the NT control, although these estimated increases did not reach statistical significance. For CXCL9, all three NT and all three p50 samples were undetectable and assigned the LLoD value for analysis. We determined that CXCL9 secretion was increased at least 128‐fold in the absence of STAT6 and 46‐fold in the absence of both p50 and STAT6. These patterns of protein secretion were similar at 48 h, although secreted TNFα levels were significantly higher (*P* = 0.04) in the p50 + STAT6 compared with the NT samples at that time (data not shown). Taken together, qRT‐PCR confirmed selected differential gene expression identified by RNA sequencing, while Luminex analysis demonstrated corresponding changes in protein secretion. These findings support the conclusion that STAT6 and p50/STAT6 deficiency promote a proinflammatory macrophage program despite exposure to IL‐4.

Because pathway analysis of the RNA‐seq data suggested altered antigen presentation pathways in p50/STAT6‐deficient macrophages, we next evaluated expression of HLA‐ABC (MHCI), HLA‐DR (MHCII), and the associated costimulatory molecules CD80 and CD86 by flow cytometry. Gene‐edited human macrophages generated from CD34^+^ cells were either left unpolarized (M0) or polarized for 24 h with IL‐4 (M2) or IFNγ (M1) before analysis. Expression of MHCI, MHCII, CD80, and CD86 was largely preserved across NT, *NFKB1*, *STAT6*, and *NFKB1* + *STAT6* gene‐edited macrophages under M0, M1, and M2 polarization conditions (Fig. 9). In the presence of IL‐4, approximately 97% of macrophages expressed MHCI and 90–93% expressed MHCII regardless of genotype. However, p50/STAT6‐deficient macrophages exhibited reduced MHCI and MHCII GMFI (24% and 22%, respectively), relative to NT macrophages. Expression of the costimulatory molecules CD80 and CD86 was also evident in the large majority of macrophages (83–87% and 81–87%, respectively) across all genotypes. However, STAT6‐ and p50/STAT6‐deficient macrophages exhibited reduced CD80 (8–11%) and CD86 (24–30%) GMFI under IL‐4 conditions. Thus, despite transcriptional alterations in antigen presentation pathways identified by RNA‐seq, p50/STAT6‐deficient macrophages maintained the frequency of MHCI‐, MHCII‐, CD80‐, and CD86‐expressing cells, with only modest reductions in GMFI in the presence of IL‐4.

**Fig. 9:**
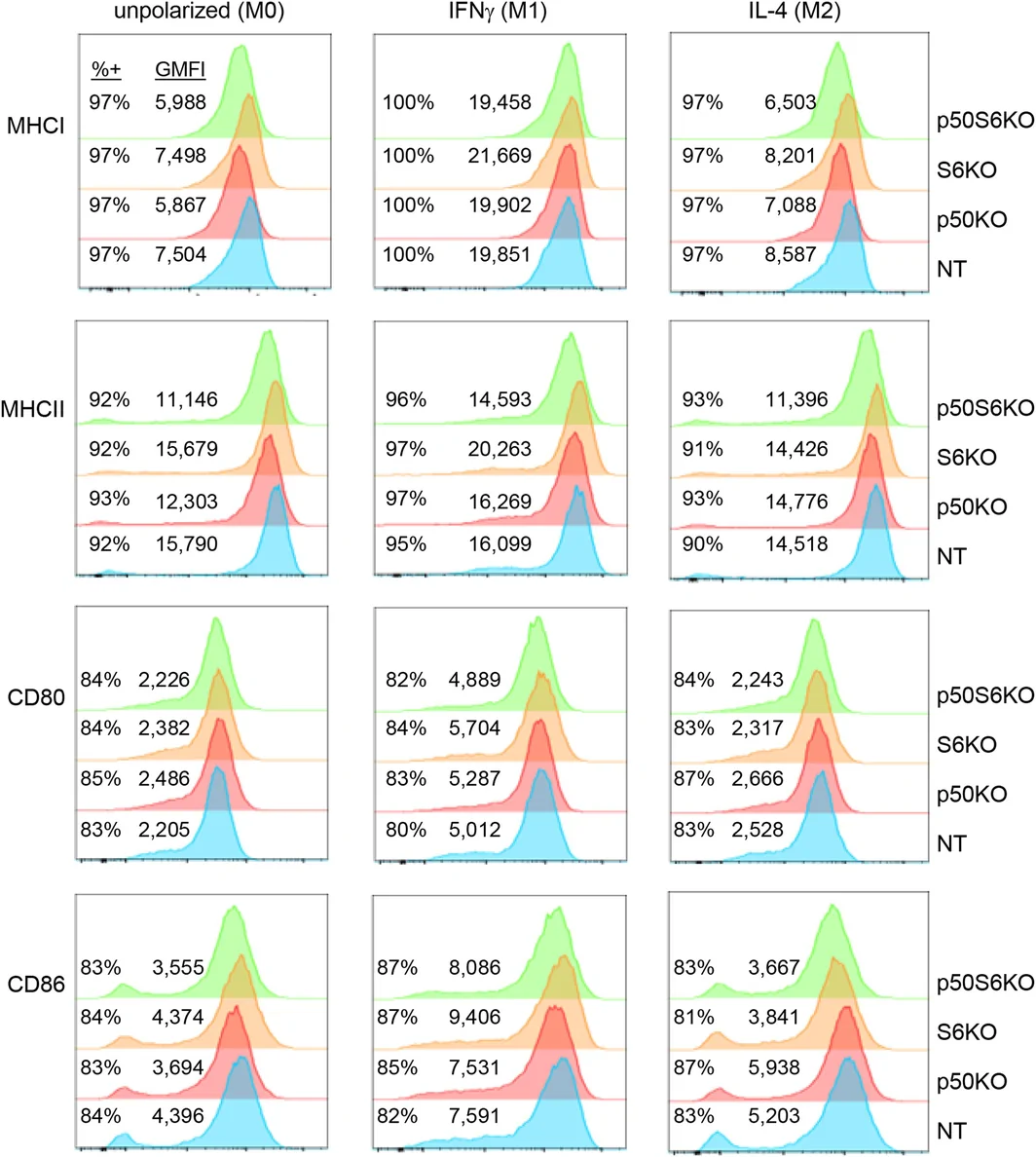
MHCI, MHCII, CD80, and CD86 expression on human macrophages lacking p50, STAT6, or both p50 and STAT6. Human marrow CD34^+^ cells from a single donor were gene‐edited, expanded, and differentiated to macrophages by culture in M‐CSF for 5 days followed by human AB serum for 7 days. They were then either maintained unpolarized (M0) or M1‐ or M2‐polarized for 24 h using IFNγ or IL‐4. Surface MHCI, MHCII, CD80, and CD86 were evaluated using flow cytometry (*n* = 1). %− positive (%+) cells and geometric mean fluorescence indices (GMFI) are shown. NT—nontargeted gene‐editing.

To further assess whether gene editing altered macrophage effector function relevant to antitumor activity, we next evaluated antibody‐dependent tumor cell phagocytosis. Phagocytosis assays were performed using gene‐edited macrophages. Following 24 h of M0, M1, or M2 polarization, macrophages were cocultured with GD2 antibody‐opsonized SK‐N‐DZ human neuroblastoma cells labeled with CFSE. Tumor cell phagocytosis by macrophages was quantified by flow cytometry analysis of CFSE^+^ macrophages. No appreciable differences in GD2 antibody‐mediated phagocytosis were observed among NT, *NFKB1*, *STAT6*, and *NFKB1* + *STAT6* gene‐edited macrophages under M0, M1, or M2 conditions (Fig. 10A,B). Comparable phagocytic activity across genotypes is consistent with preserved FcγR1 expression and indicates that Fcγ receptor‐dependent tumor cell phagocytosis is maintained in the absence of p50 and/or STAT6.

**Fig. 10:**
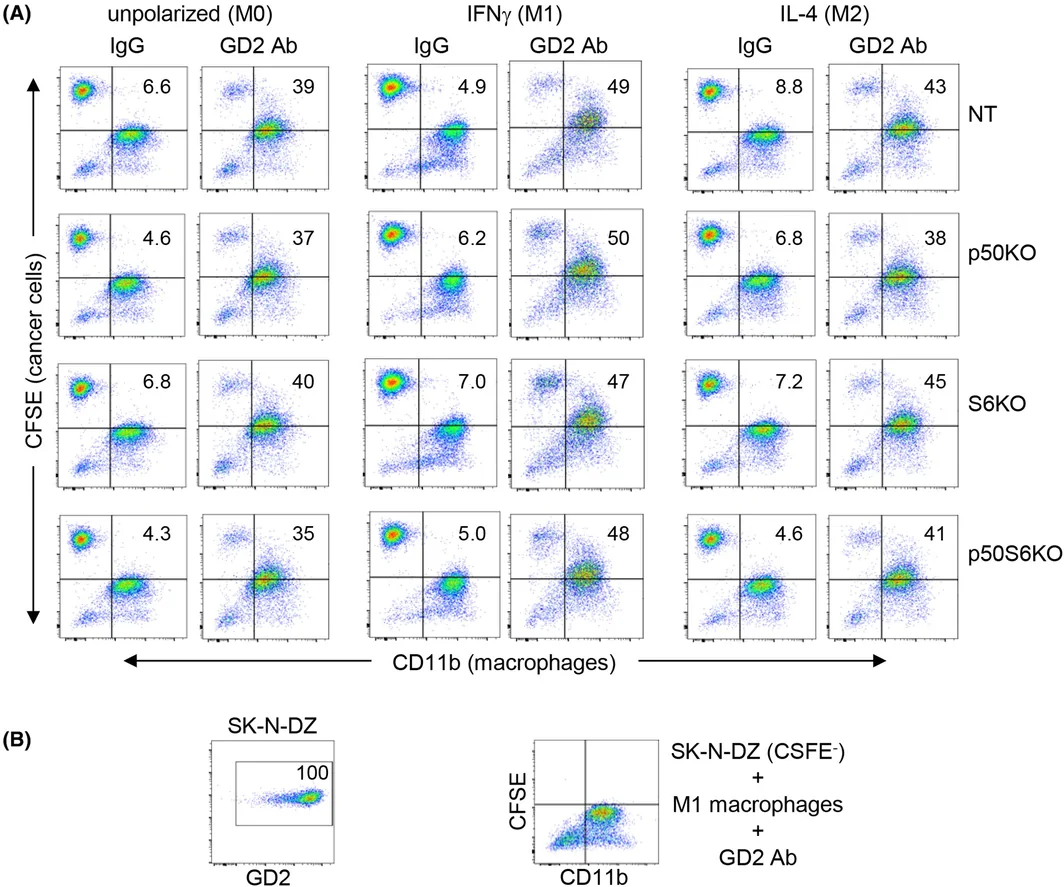
Phagocytosis by human macrophages lacking p50, STAT6, or both p50 and STAT6. (A) Human marrow CD34^+^ cells from a single donor were gene‐edited, expanded, and differentiated to macrophages by culture in M‐CSF for 5 days followed by human AB serum for 7 days. They were then either maintained unpolarized (M0) or M1‐ or M2‐polarized for 24 h using IFNγ or IL‐4, followed by coculture for 3 h with CFSE‐labeled SK‐N‐DZ human neuroblastoma cells that had been incubated with GD2 antibody (Ab) or isotype human IgG1 control. The proportions of macrophages that have phagocytosed cancer cells (CD11b^+^CFSE^+^) are shown (*n* = 1). NT—nontargeted gene‐editing. (B) Expression of GD2 on SK‐N‐DZ cells was assessed by flow cytometry (left, *n* = 1). Control for establishing flow cytometry gates (right).

## 4 Discussion

The objective of this study was to establish a clinically relevant platform for engineering proinflammatory human myeloid cells that resist tumor‐associated immunosuppressive signals. We developed a robust serum‐free expansion protocol for human bone marrow‐derived CD34^+^ hematopoietic stem and progenitor cells that enabled sustained proliferation while preserving the CD34^+^CD38^−^ stem/progenitor phenotype. We also identified a method to generate CD14^+^ monocytic cells from expanded CD34^+^ HSPCs. Using optimized CRISPR/Cas9 ribonucleoprotein delivery, we achieved near‐complete (≥ 96%) and reproducible deletion of *NFKB1*, *NFKB2*, and *STAT6*, individually or in combination, without impairing subsequent myeloid differentiation. Edited cells efficiently generated CD11b^+^CD14^+^ immature myeloid cells and retained expression of IL4R, IFNγR, and FcγR1, supporting their capacity to respond to cues within the tumor microenvironment and their compatibility with antibody‐based targeting strategies. We previously demonstrated that antibody‐ or CAR‐mediated targeting facilitates *in vitro* phagocytosis and tumor localization of p50‐deficient murine myeloid cells [37]. Consistent with murine data, our gene‐edited human macrophages maintained antibody‐dependent tumor cell phagocytosis across genotypes, indicating preservation of Fcγ receptor‐mediated effector function following deletion of p50 and/or STAT6. Taken together, these findings establish a scalable and clinically adaptable strategy for generating targeted, gene‐edited human immature myeloid cells for adoptive immunotherapy.

Using this platform, we demonstrated that deletion of STAT6, or combined deletion of NF‐κB p50 and STAT6, confers a robust proinflammatory phenotype in human macrophages exposed to IL‐4, a canonical M2‐polarizing and immunosuppressive cytokine [38]. Both STAT6‐ and p50/STAT6‐deficient macrophages exhibited strong enrichment of inflammatory Hallmark pathways, marked induction of M1‐associated transcripts, suppression of IL‐4‐induced M2 genes, and increased secretion of proinflammatory mediators, including IL‐6 and CXCL10. These results are consistent with prior observations in murine *Stat6* ^−/−^ bone marrow‐derived macrophages [18] and support a central role for STAT6 in mediating IL‐4‐driven suppressive polarization.

Surprisingly, deletion of p50 alone, or p50 together with its homolog p52, did not broadly activate inflammatory pathways under IL‐4 polarizing conditions, although increases in TNFα and IL12β transcripts (and protein) were observed, with a trend toward increased IL‐6 expression. This finding was unexpected given multiple reports demonstrating that p50 restrains M1 transcriptional programs in murine models. Saccani et al. showed that p50 is overexpressed in murine tumor‐associated macrophages and suppresses proinflammatory gene expression when TAM are stimulated *ex vivo* with LPS [4]. We have reported that tumor macrophages isolated from syngeneic orthotopic glioblastomas growing in p50^−/−^ mice express increased M1 mRNAs and reduced M2 mRNAs compared to macrophages from tumors in WT mice [12] and have demonstrated efficacy of murine IMC lacking p50 against various syngeneic tumors in mice [14, 15]. These reports suggest that the impact of p50 loss may depend on the inflammatory context and additional signals present within the tumor microenvironment. IL‐4 is widely used to model alternatively activated macrophages and recapitulates a major suppressive axis present in many solid tumors, but it does not capture the full complexity of the tumor microenvironment. Thus, the comparatively modest transcriptional effects observed in human macrophages lacking p50 under IL‐4 polarization may reflect the absence of additional tumor‐derived signals that induce p50's repressive effects *in vivo*.

Detailed analysis of the proinflammatory M1 gene set revealed that STAT6 deletion alone induced a strong proinflammatory program, whereas dual p50/STAT6 deletion produced a distinct pattern in which some M1‐associated transcripts were attenuated while others were further increased. These data indicate that both STAT6‐deficient and p50/STAT6‐deficient macrophages adopt a net proinflammatory phenotype in the presence of IL‐4, but the specific transcriptional architecture differs between single and dual deletions.

In addition to promoting inflammatory reprogramming, STAT6 deletion was associated with enrichment of cell cycle‐related programs. Despite these signatures, STAT6‐deficient CD34^+^ progenitors did not exhibit enhanced expansion, suggesting that this enrichment reflects transcriptional rewiring rather than increased proliferative capacity. Importantly, these changes did not impair macrophage differentiation or inflammatory cytokine production. In contrast, alterations in antigen presentation pathways were most prominently associated with loss of p50. GO analysis revealed reduced enrichment of MHC class II–related pathways in p50‐deficient macrophages, with combined p50/STAT6 deletion additionally associated with decreased MHC class I pathway enrichment. The functional significance of these transcriptional changes remains uncertain given the context‐dependent nature of p50‐mediated transcriptional regulation; indeed, loss of p50 has been reported to enhance neoantigen presentation capacity in certain settings [11]. To further interrogate this finding, expression of the key antigen presentation and costimulatory molecules MHCI, MHCII, CD80, and CD86 was assessed by flow cytometry. Although p50/STAT6‐deficient macrophages exhibited modest reductions in GMFI of MHCI, MHCII, CD80, and CD86 under IL‐4 conditions, the frequency of cells expressing these proteins was maintained across genotypes.

Collectively, the results presented herein demonstrate that deletion of STAT6, alone or in combination with p50, generates human macrophages capable of resisting IL‐4‐mediated immunosuppression, while highlighting distinct transcriptional consequences that may influence their therapeutic potential. As such, we are unable to predict whether targeting STAT6 alone or combining p50/STAT6 deletion will provide superior therapeutic efficacy, and both approaches will require direct clinical evaluation.

In murine tumor models, the antitumor efficacy of p50‐deficient immature myeloid cells is T‐cell–dependent [14, 15]. Although we did not see efficacy of p50‐IMC when used to target an MHC‐mismatched cancer in MHC‐mismatched mice [15], it has not been established whether direct antigen presentation by transferred cells is required for therapeutic activity or whether enhanced secretion of proinflammatory mediators is sufficient to activate endogenous antigen‐presenting cells and amplify adaptive immunity. Consistent with this latter possibility, both STAT6‐ and p50/STAT6‐deficient macrophages produced high levels of CXCL10; however, the maximal concentrations measured in our cultures were below the threshold reported to induce human T‐cell chemotaxis *in vitro* [39]. Whether higher local concentrations within tumors, combined effects of multiple inflammatory mediators, or interactions with endogenous immune cells contribute to T‐cell recruitment and activation remains to be determined. In addition, future studies using cocultures of MHC‐matched gene‐edited human macrophages, human T cells, and human cancer cells will determine whether differences in antigen presentation pathways translate into functional effects on MHC‐mediated T‐cell activation. If proinflammatory cytokine secretion proves to be the predominant mechanism for STAT6‐IMC or p50/STAT6‐IMC efficacy in patients, these cells could be modified to evade host immune rejection, forming the basis of a broadly applicable, off‐the‐shelf allogeneic immunotherapy.

Human p50‐IMC efficacy likely requires adoptive transfer of sufficient cell numbers. In mice, when we compared efficacy of 5FU followed by three injections of 1 × 10^7^ or 4 × 10^6^ murine p50‐IMC, syngeneic prostate cancer tumor response was reduced at the lower cell dose [14]. Using our platform, CD34^+^ cells can expand 10 000‐fold over 30 days, with an even greater rate of proliferation upon transfer to M‐CSF. Efficient expansion, together with tumor‐targeting strategies such as tumor‐directed antibodies or transduction with a tumor‐specific chimeric antigen receptor (CAR), may reduce the number of STAT6‐ or p50/STAT6‐IMC required for therapeutic efficacy. Additional combinatorial approaches, including T‐cell checkpoint blockade or therapeutics that augment tumor cell phagocytosis, such as anti‐CD47 antibody [33], may further enhance benefit. Collectively, scalable expansion, capacity for targeted tumor engagement, and proinflammatory reprogramming define a framework for the clinical translation of gene‐edited human myeloid cell therapies for cancer.

## 5 Conclusion

In summary, we established a scalable platform for generating gene‐edited human myeloid cells from CD34^+^ hematopoietic stem and progenitor cells. Loss of STAT6, alone or together with NF‐κB p50, promotes a global proinflammatory phenotype and reduces IL‐4‐induced immunosuppressive gene expression while preserving myeloid differentiation and antibody‐dependent tumor cell phagocytosis. Although both genotypes resist IL‐4‐mediated immunosuppression, their distinct transcriptional profiles highlight potentially important differences in therapeutic function. Loss of p50 induced expression of several proinflammatory cytokines in IL‐4 and was previously shown to slow tumor growth in mice. These findings support STAT6 and p50/STAT6 deletion as approaches for engineering IL‐4‐resistant human myeloid cells for adoptive cell immunotherapy against human malignancies.

## Conflict of interest

A.D.F. is a named inventor on a US patent application entitled ‘NF‐κB p50 Deficient Immature Myeloid Cells and Their Use in Treatment of Cancer’ and provisional patent application ‘Proinflammatory Immature Myeloid Cells and Their Use in Treatment of Cancer’ filed by the Johns Hopkins University. Under a license agreement between Vita Therapeutics, Inc. and the Johns Hopkins University, Dr. Alan D. Friedman and the University received royalty distributions related to the technology covered by the forementioned US patent application and described in these publications [14, 15]. Dr. A. D. Friedman has also acted as an unpaid member of the Scientific Advisory Board to Vita Therapeutics, Inc. This arrangement has been reviewed and approved by Johns Hopkins University in accordance with its conflict of interest policies.

## Author contributions

TB and ADF contributed to the conception and design of the work. TB and ADF performed the experiments. TB and ADF analyzed and interpreted the data. ADF wrote the manuscript. TB analyzed and revised the manuscript.

## Supporting information


**Fig. S1.** M‐CSF versus GM‐CSF for induction of monocytic maturation of human CD34^+^ cells.

**Fig. S2.** DNA sequence analysis after *NFKB1*, *NFKB2*, and *STAT6* gene‐editing.

**Fig. S3.** Initial expansion of non‐nucleofected, nontargeted (NT), and p50KO human CD34^+^ cells.

**Fig. S4.** MCSFR expression in NT, p50KO, STAT6KO, p50/STAT6KO, and p50/p52KO human CD34^+^ cells.

**Fig. S5.** CD34/CD38 expression in NT, p50KO, STAT6KO, p50/STAT6KO, and p50/p52KO human CD34^+^ cells after their transfer to M‐CSF.

**Fig. S6.** CD11b, CD14, and CD34 geometric mean fluorescence index in NT, p50KO, STAT6KO, p50/STAT6KO, and p50/p52KO human CD34^+^ cells after their transfer to M‐CSF.

**Fig. S7.** Human CD34^+^ NT, p50KO, STAT6KO, p50/STAT6KO, and p50/p52KO cell populations include few lymphocytes, granulocytes, or erythroid cells after expansion or transfer to M‐CSF.

**Fig. S8.** A proinflammatory M1 Gene Set and associated pathways and an M2 Gene Set.

**Fig. S9.** M2 RNA expression by human CD34^+^ cell‐derived macrophages in IL‐4, obtained after NT, *NFKB1, STAT6*, or *NFKB1 + STAT6* gene‐editing.

**Table S1.** RT‐PCR primer pairs.

**Table S2.** RNA‐Seq gene expression data from human marrow CD34^+^ cell‐derived macrophages in IL‐4, obtained after NT, *NFKB1, STAT6, NFKB1 + STAT6*, or *NFKB1 + NFKB2* gene‐editing.

**Table S3.** KEGG and GO pathways for NT vs p50, p50p52, STAT6, and p50/STAT6 KO macrophages.

## Data Availability

The datasets generated and/or analyzed during the current study are available from the corresponding author upon reasonable request.
